# Disentangling physical and biological drivers of phytoplankton dynamics in a coastal system

**DOI:** 10.1038/s41598-017-15880-x

**Published:** 2017-11-20

**Authors:** Daniela Cianelli, Domenico D’Alelio, Marco Uttieri, Diana Sarno, Adriana Zingone, Enrico Zambianchi, Maurizio Ribera d’Alcalà

**Affiliations:** 1 Dipartimento di Scienze e Tecnologie, Università degli Studi di Napoli Parthenope, Centro Direzionale di Napoli – Isola C4, 80143 Naples, Italy; 2 ISPRA – Istituto Superiore per la Protezione e la Ricerca Ambientale, Via Vitaliano Brancati 60, 00144, Rome, Italy; 3 CoNISMa (Consorzio Nazionale Interuniversitario per le Scienze del Mare), Piazzale Flaminio 9, 00196 Rome, Italy; 4 Stazione Zoologica Anton Dohrn, Villa Comunale, 80121 Naples, Italy; 5 ISAC-CNR, Via Fosso del Cavaliere 100, 00133 Rome, Italy

## Abstract

This proof-of-concept study integrates the surface currents measured by high-frequency coastal radars with plankton time-series data collected at a fixed sampling point from the Mediterranean Sea (MareChiara Long Term Ecological Research site in the Gulf of Naples) to characterize the spatial origin of phytoplankton assemblages and to scrutinize the processes ruling their dynamics. The phytoplankton community generally originated from the coastal waters whereby species succession was mainly regulated by biological factors (life-cycle processes, species-specific physiological performances and inter-specific interactions). Physical factors, e.g. the alternation between coastal and offshore waters and the horizontal mixing, were also important drivers of phytoplankton dynamics promoting diversity maintenance by i) advecting species from offshore and ii) diluting the resident coastal community so as to dampen resource stripping by dominant species and thereby increase the numerical importance of rarer species. Our observations highlight the resilience of coastal communities, which may favour their persistence over time and the prevalence of successional events over small time and space scales. Although coastal systems may act differently from one another, our findings provide a conceptual framework to address physical–biological interactions occurring in coastal basins, which can be generalised to other areas.

## Introduction

Changes in phytoplankton assemblages at a specific site, such as at Long Term Ecological Research (LTER) ones, may be difficult to interpret, because they result from two main, potentially interplaying key factors, namely: physical transport and biological processes, which are classically indicated as ‘allogenic’ and ‘autogenic’ factors, respectively^1,2^.

Physical transport may imply either the mixing or the replacement of distinct plankton assemblages. Biology, conversely, determines the local gain and loss of individuals *via* a myriad of processes, such as cell division and life cycle shifts^3^, inter-specific differences in growth rates due to different physiological behaviour^4^, as well as positive and negative inter-specific interactions, which ultimately result in the succession of species^4^. The interplay of these different processes generates potential biases in interpreting the mechanisms underlying the waxing and waning of phytoplankton blooms^5^, some of which are toxigenic and, thus, noxious to human health^6^. This methodological problem is further aggravated in coastal and relatively shallow areas, where horizontal gradients are stronger^7,8^. As a result, the interpretation of the complex spatial-temporal dynamics of plankton communities at LTER sites is often oversimplified, especially in case of single-point sampling sites.

A possible way to overcome these limitations is the integration of the ecological information with that of the surface circulation. The latter may be described with an unprecedented accuracy, resolution and synopticity through land-based remote observations^9^, such as those provided by high frequency coastal radars (HFRs)^10^. Protocols for the estimation of hourly surface velocity maps from HFRs are becoming increasingly robust^11^. Not only do HFR data allow following the progress of specific events, but they can also be used to analyze the coastal transport processes by means of consolidated Lagrangian modelling approaches^12–14^, such as the backtracking model simulations. These have been widely applied in the atmospheric field^15,16^ to pinpoint the origin of pollutants from multiple emission sites, whereas they have been poorly employed in the marine environment^13,17^.

In this study, we explored and discussed the possibility of combining the ecological observations performed at the reference site LTER-MareChiara (LTER-MC) in the Gulf of Naples (GoN; Mediterranean Sea), with the continuous mapping of surface currents by using a CODAR HFR over the same geographic area. This proof-of-concept investigation was intentionally focused on a single reference year and aimed at paving the way to more systematic studies on the link between coastal circulation and plankton dynamics. The high resolutions of both physical and ecological observations allowed us to detect short-term changes in species’ abundance and to relate them to either horizontal transport or biological processes intrinsic to the phytoplankton community, in different seasons and phases of coastal circulation.

### Physical and ecological contexts and analysis rationale

**Figure 1:**
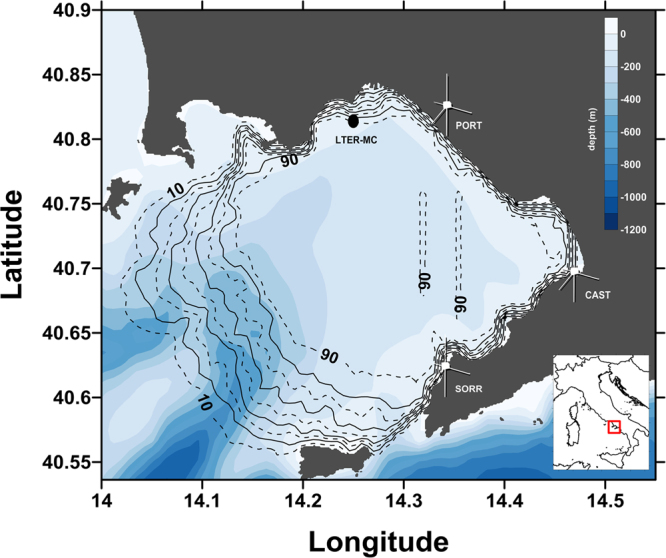
Map of the Gulf of Naples (GoN) showing the location of the three HFR stations (antenna icon) with radar coverage percentage and the location of the LTER-MC station (black dot). Created with Surfer Ver. 10.7.972 (64-bit); www.goldensoftware.com. Coastline data: NOAA National Geophysical Data Center, Coastline extracted: WLC (World Coast Line), Date Retrieved: 01 April, 2015, http://www.ngdc.noaa.gov/mgg/shorelines/shorelines.html; bathymetric data from Amante and Eakins (2009) [Amante, C. and B. W. Eakins (2009). “ETOPO1 1 Arc-Minute Global Relief Model: Procedures, Data Sources and Analysis.” Access date: 08 September 2011, from doi:10.7289/V5C8276M].

The GoN is located along the western coast of Italy in the Mid Tyrrhenian Sea (Western Mediterranean Sea) (Fig. 1), with the densely inhabited urban area of Naples set on its northern/north-eastern side. This marginal, semi-enclosed basin presents complex hydrological and dynamical features due to its intricate bottom topography and the alternation of different water masses during the year^10,18,19^. The GoN hosts two coexisting subsystems: one with relatively oligotrophic, offshore (open water) characteristics; and the other with typical moderately eutrophic, coastal features^20–22^. Previous studies have revealed that the boundary between the two subsystems is subject to a seasonal shift, being closer to the coast in summer and displaced offshore in winter^20,21,23^; the surface circulation and the associated transport processes show a marked seasonal signature as well^10,14,23–25^.

The LTER-MC sampling site (40.81°N, 14.25°E) is located two nautical miles away from the coast in the proximity of the 75 m isobath, in an area influenced by coastal conditions over most of the year^26^. At LTER-MC, basic hydrographic parameters, such as temperature, salinity, chlorophyll *a* (chl *a*) as well as species composition of plankton communities were determined bi-weekly since 1984 and on a weekly base since 1994^26^. A specific feature of phytoplankton in this system is the occurrence of intense diatom blooms in the late-spring and summer, besides the typical seasonal peaks in late-winter/spring and autumn^21,22,26,27^: these blooms sustain the plankton productivity in the GoN during summer^28^. Furthermore, several phytoplankton species are characterized by consolidated recurrent patterns in their annual occurrence^29^. Based on its geographic position, setting in the middle between offshore and coastal dominions, the LTER-MC site is representative of a wider area, in particular of the north-eastern part of the GoN^20,22,26^. The coastal system thus potentially extends beyond the above-mentioned sampling site, although the degree of its extension may depend on physical factors driven by seasonally specific dynamics.

Our study is primarily oriented towards reconstructing the transport processes carrying water masses and plankton particles to the LTER-MC station on a weekly basis (i.e., the time-scale of regular plankton samplings at that site), by using HFR data for backtracking simulations. The phytoplankton composition was examined on a weekly time scale to disentangle physically- from biologically-driven community modifications. Our analyses were conducted for the reference year 2009 and included the following conceptual steps, whose methodological details can be found in the Methods section and in the Supplementary Tables and Note.

1. As a first step the 2009 annual and seasonal regimes of HFR-derived surface currents at LTER-MC were analyzed. Second, backtracking simulations were conducted using as advection field the HFR data in the opposite time and direction. Virtual phytoplankton patches (VPPs hereinafter) were released at LTER-MC on the dates of the weekly oceanographic campaigns and tracked backward in time (up to 96 h earlier) to their zone of origin. This simulation time was selected based on preliminary comparative tests; it assumes a phytoplankton growth rate of one division per day and represents a good compromise to appropriately resolve the scales of both physical transport and biological processes.
2. In order to classify the origin zones of the water masses reaching LTER-MC, we identified eight main sectors in the GoN that radiated from the LTER-MC site based on specific geographic and environmental features (urban areas, presence of harbours, etc.): sectors 1–4 and 8 were associated with the coastal areas extending up to 200 m from the coastline, whereas sectors 5–7 defined the offshore origin areas. Based on these sectors, we categorized the VPPs arriving at LTER-MC as originating from either waters close to the GoN coastline, offshore waters, or from both the directions at the same time (hereinafter, coastal, offshore and mixed origin, respectively).
3. We then reconstructed: i) the distribution of the origin zones of VPPs for each *in situ* sampling date, ii) the time taken by VPPs to reach LTER-MC from the origin zone (first-entry time T_A_, hereinafter) and iii) an index of the spreading of VPPs (Spr) (see Supplementary Note). These data allowed inferring the horizontal spreading of plankton patches directed to LTER-MC and the area that the plankton community sampled at LTER-MC presumably represents. This information ultimately revealed whether a given plankton community developed in that particular place or was transported there from elsewhere.
4. The above-mentioned descriptors of the surface dynamics were then compared with salinity, chl *a* and phytoplankton diversity data recorded at LTER-MC to: i) inter-validate the identification of surface water origin based on VPPs modelling with the ecological characterization of the reference station and ii) identify the distinct states of the plankton system based on both physical and ecological proxies.
5. In order to assess the relative importance of the two main drivers, i.e. the physical and the biological ones, in determining the development in time of phytoplankton assemblages, five examples of temporal sequences were analysed with hierarchical clustering. For each sequence, different phytoplankton samples were aggregated based on mutual similarity (Q-mode), whereas species were aggregated based on shared trends over time (R-mode analysis). The examples were selected from among sequences of persistent coastal water conditions or hydrographic changes. The aim was to distinguish sharp changes in phytoplankton composition caused by physical drivers (e.g., the advection of species from offshore sectors or dispersion of coastal species) from successional events predominantly driven by biological processes (e.g., changes in growth rates, formation/germination of resting stages and inter-specific interactions). The impact of surface dynamics on the diversity of phytoplankton was analysed across the five examples using the ratio between phytoplankton diversity (Fisher’s alpha) and biomass (chl *a*).

## Results and Discussion

### Physical dynamics: surface currents and VPPs origin

**Figure 2:**
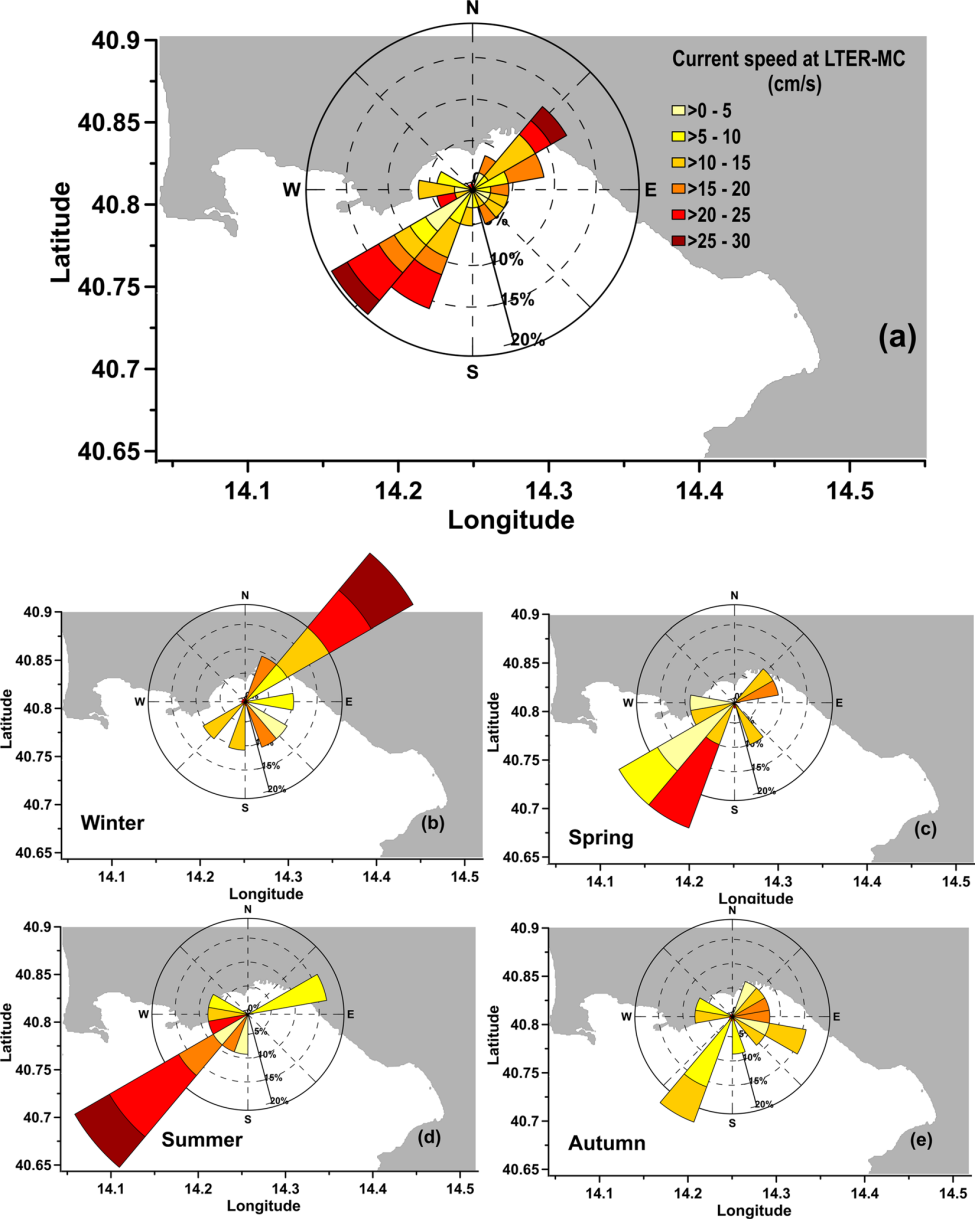
(**a)** Annual regime of HFR surface currents at LTER-MC; (**b–e)** Seasonal regime of HFR surface currents at LTER-MC. Each lobe represents the frequency with which the current flows from a given direction, whereas the angular regions separate data into current speeds. Azimuthal bins indicate the direction from which the current is coming. Created with Grapher Ver. 9.6.1001 (64-bit): www.goldensoftware.com. Coastline data: NOAA National Geophysical Data Center, Coastline extracted: WLC (World Coast Line), Date Retrieved: 01 April, 2015, http://www.ngdc.noaa.gov/mgg/shorelines/shorelines.html.

The annual and seasonal analyses of surface currents based on HFR data are presented in Fig. 2. The surface current fields were primarily forced by the local wind regime^23^, as typical for the GoN^10,14,23–25^. On an annual scale, the predominant and most intense currents alternated their direction between the NE and SW, with maximum speeds in the range of 15–25 cm s^−1^ (Fig. 2a). Currents from the SE direction were much less frequent and less intense (≤10 cm s^−1^). The seasonal analysis of instantaneous currents at the sampling times showed that the two principal directions of origin (from the NE and SW) were associated with the relatively more intense currents occurring during the winter and summer months, respectively, with maximum speeds in the range of 25–30 cm s^−1^ (Fig. 2b and d). In the same months, currents from SE and NW were much less frequent and less intense (<15 cm s^−1^). During the spring and autumn seasons, the directions of origin were more variable. In particular, during spring the strongest currents (5–20 cm s^−1^) came from the west-southwest (WSW) direction, whereas in autumn the current intensities were generally weaker (<15 cm s^−1^) and most frequently from the SW and SE (Fig. 2c and e).

**Figure 3:**
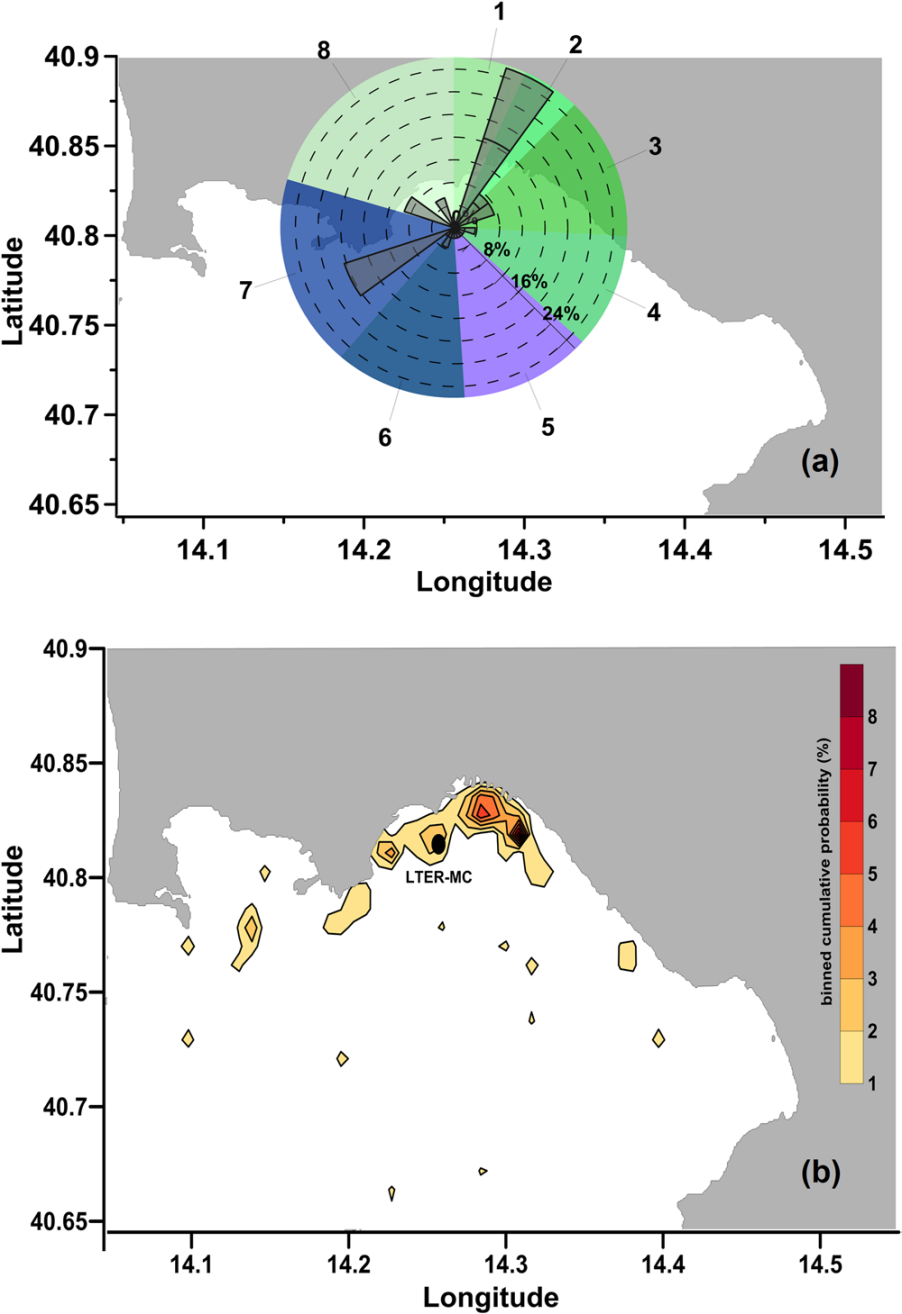
(**a)** Annual distribution of VPPs origin area: sectors indicate the direction from which VPPs arrive at LTER-MC as resulting from backtracking simulations. Index sectors of VPPs origin zones in the GoN: 1-2-3-4-8 = coastal areas; 5-6-7 = offshore areas. Created with Grapher Ver. 9.6.1001 (64-bit): www.goldensoftware.com. (**b)** Binned cumulative probability of weekly VPP origin at LTER-MC. The total number of particles for each bin was calculated (cumulated over the entire set of model runs), and the relative percentage contribution was then estimated. For a proper visual representation, data were gridded using a minimum curvature method to generate the smoothest possible surface while at the same time honouring data at best. Created with Surfer Ver. 10.7.972 (64-bit); www.goldensoftware.com. Coastline data: NOAA National Geophysical Data Center, Coastline extracted: WLC (World Coast Line), Date Retrieved: 01 April, 2015, http://www.ngdc.noaa.gov/mgg/shorelines/shorelines.html.

A synthesis of the directions from which VPPs get to LTER-MC, as detected *via* backtracking forced by the HFRs data, is presented in Fig. 3a. It displays the percentage of occurrences of each VPP coming from the pre-defined eight sectors: this elaboration indicates that, during the year 2009, the surface water (and the plankton within) detected at LTER-MC mainly comes from the coastal dominion. In 30% of these simulations, the VPPs came from the harbour and the eastern littoral zone of Naples (NNE sectors 1 and 2); in 10% of the simulations, the VPPs came from the area of the seaside promenade (sector 8); in 31.4% of the simulations, VPPs came from the offshore zone (sectors 5–7) and prevalently from the WSW direction (20%; sector 7). As a complement to this analysis, we plotted the spatial distribution of the VPPs origin zones (cumulative annual percentage) (Fig. 3b). This elaboration provided a view of the geographical arrangement of the origin zones of VPPs at the beginning of the transport process toward LTER-MC. This plot emphasized that, on a yearly basis, VPPs mostly originated from the northern sub-basin of the GoN (i.e., from 40.75°N) and only occasionally got to LTER-MC from farther south. This observation was consistent with a latitude-dependent surface circulation pattern described in a previous study^23^ and is associated with the presence of basin- and sub-basin-scale structures of the flow field.

**Figure 4:**
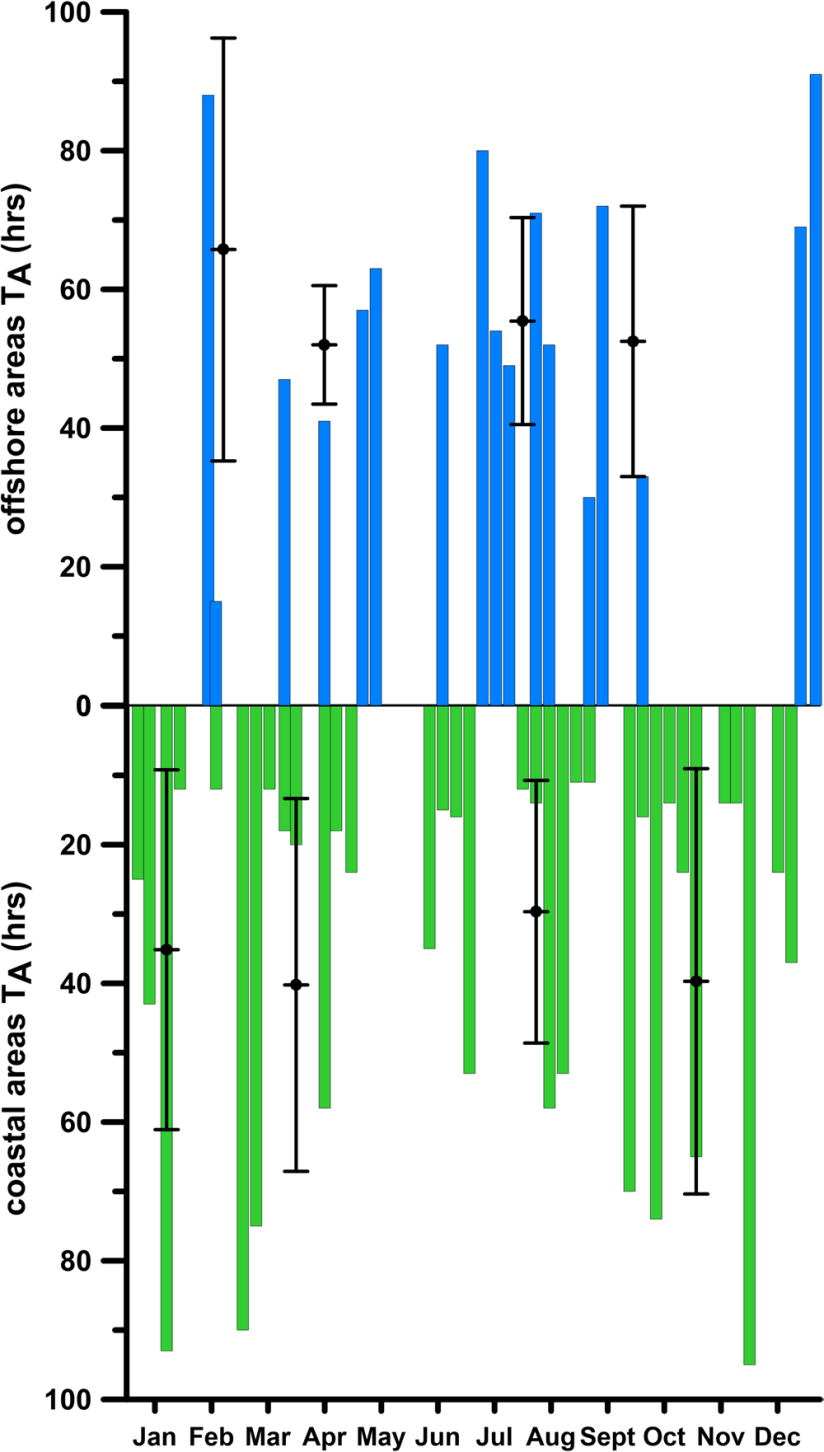
The first-entry time (T_A_) taken by VPPs to arrive at LTER-MC from the origin zone. Bars represent VPPs coming from the coastal areas (green bars) or from offshore areas (blue bars). Black dots and error bars inside the figure indicate the average and standard deviations values for each season.

While the former elaborations indicated the geographic origin of VPPs, the T_A_ analysis provided the temporal scales characterizing the process of their transport. Plots in Fig. 4 indicate the number of hours needed by VPPs to reach LTER-MC from their origin zones (i.e., the inverse of the time needed to ‘backtrack’ VPPs from LTER-MC to their origin zones). When the surface water arrived to LTER-MC from the coastal dominion (green bars in Fig. 4), VPPs reached LTER-MC within approximately 36 h on average. Shorter T_A_ from the coast (approximately 12 hours) occurred frequently during the spring and summer months when the wind breeze regime caused the daily rotation of the surface current field, with the alternation of coastward and offshore currents^10,14,23,24^. Higher T_A_ values were recorded during the winter, when VPPs took up to 95 h to reach LTER-MC from the coastal areas (Fig. 4, lower panel). When the surface water originated from the offshore areas, the average T_A_ for VPPs reaching LTER-MC was higher (approximately 56 h) than for the coastal origin (see above and Fig. 4, blue bars). The minimum T_A_ values (30 h) were recorded during the spring and summer months (due to the breeze regime effect), whereas winter T_A_s (up to 91 h) were similar to those of the coastal origin of VPPs. These findings are similar to previous studies that reported how surface dynamics driven by different local and remote forcing affect the residence times of particles released in the neighbourhood of LTER-MC^24,25^.

### Integrating physical and ecological observations

**Figure 5:**
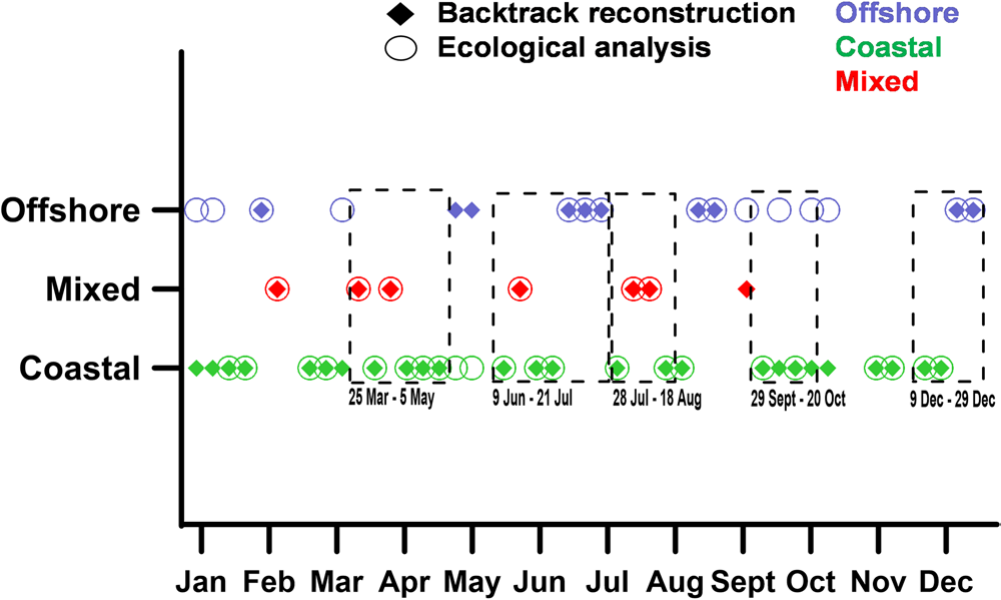
Comparison among backtrack Lagrangian reconstruction and ecological analysis based on salinity and chlorophyll *a* data obtained through weekly sampling at LTER-MC. Green = VPPs originating from coastal areas; blue = VPPs originating from offshore areas; red = VPPs originating partly from coastal and partly from offshore areas. The ecological classification was obtained using cluster analysis of water samples based on salinity and chlorophyll data, which are available in Table S1. Dates highlighted by dotted boxes indicate the five time periods analysed in detail in Figs 7–8.

Results of the above described Lagrangian analyses were compared with the ecological characterization of water masses at the reference site LTER-MC based on cluster analyses using the values of salinity and chl *a* in accordance with an earlier report^30^. In 79% of these cases (Fig. 5), backtracking and cluster analyses characterized the water masses in the same way. The mismatches observed were more frequent for waters recognized as of offshore origin. In seven cases, backtracking indicated a mixed origin of surface waters; in six of them, salinity and chl *a* grouped in a cluster with parameter values intermediate between those typically scored for coastal and offshore waters.

**Figure 6:**
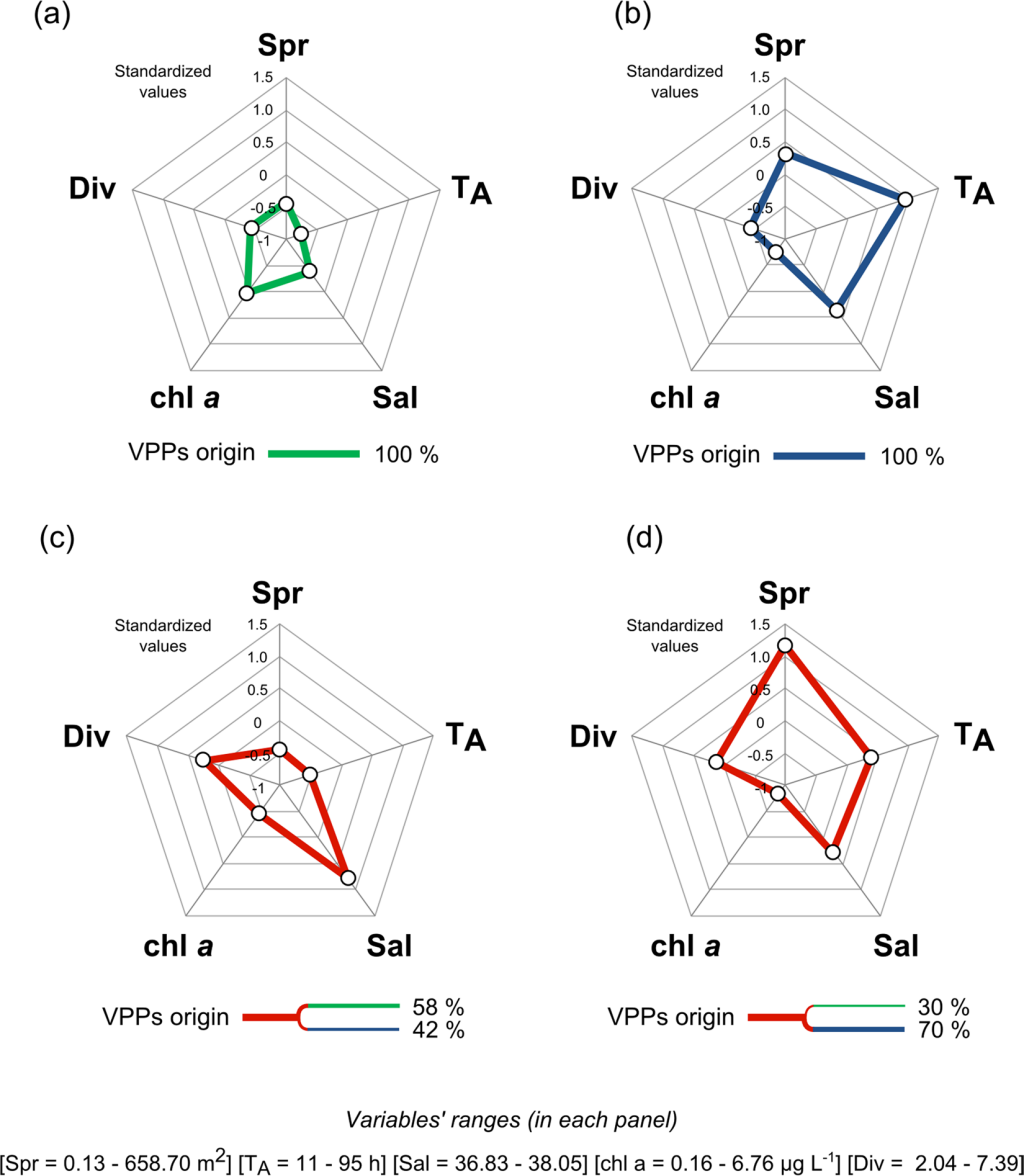
Different modes of coupled physical and ecological functioning in the GoN. (**a**) Green (coastal) phase; (**b**) blue (offshore) phase; (**c**) type 1 mixture; (**d**) type 2 mixture. Lines in each radar plot have a specific colour depending on origin zone of surface water: green and blue marked lines indicate either coastal or offshore phases, defined based on backtracking analysis; red lines indicate mixtures of coastal and offshore water masses. In all panels, ‘Spr’ is the index of spreading (see Methods and Supplementary Note), ‘T_A_’ is the first-entry time of water at LTER-MC, ‘Sal’ is salinity, ‘Div’ is the Fisher’s alpha diversity, ‘chl *a*’ is chlorophyll *a*. Radar plots were derived from standardized data for each variable. Units of measurements of each variable are presented at the bottom of the figure along with variables’ ranges in the dataset analysed.

We integrated the information on the zones of origin of water masses (i.e., coastal, offshore or mixed),T_A_ and the VPPs spreading (see also Supplementary Note) with salinity, chl *a* and phytoplankton diversity recorded at LTER-MC at the same time of simulations. In order to enhance the comparability among the above-mentioned variables (characterized by fairly different numerical ranges), standardized data were used and combined in radar plots (Fig. 6). From this dataset integration, four main states of the system based on physical and ecological proxies were identified (Fig. 6): a) green (coastal) phase, b) blue (offshore) phase, c) type 1 mixture, and d) type 2 mixture. Green phases (Fig. 6a) were characterized by VPPs coming only from coastal waters with a lower salinity (originating mainly from sectors 1–2 in Fig. 3a) and showing a relatively high geographical homogeneity (i.e., low spreading values, Supplementary Note). The coastal VPPs got to LTER-MC in a short time (i.e., lowest T_A_) and sustained the high chl *a* levels detected *in situ*. Opposite conditions were present during the blue phases (Fig. 6b), when VPPs originated only from offshore (mainly from sectors 5–7, Fig. 3a).

In addition the 7 mixed cases, where VPPs came to LTER-MC partly from the coast and partly from offshore, were all events of intrusion of offshore waters in the midst of long-lasting coastal phases (Fig. 5). We distinguished between type 1 and 2 mixtures (Fig. 6c and d). In type 1 mixture (Fig. 6c), when the VPP origin was almost half coastal and half offshore (58% and 42%, respectively), offshore waters came only from the western sectors (sector 6–7 in Fig. 3a), and spreading and T_A_ were comparable to those of the coastal phases (Fig. 6a). In type 2 mixture (Fig. 6d), spreading and T_A_ were higher than in type 1 (Fig. 6c). This condition was determined by a stronger water advection from the outer sector of the GoN (i.e., sector 5–7 in Fig. 3a), which involved a larger offshore area than in type 1 mixture. Type 2 mixture showed relatively lower chl *a* levels than type 1, due to the relatively lower contribution of coastal waters.

As for single species dynamics, the switches to the green states were generally characterised by an increase of diatoms, among which the small-sized and non-colonial ones (e.g., *Chaetoceros tenuissimus*, *Chaetoceros throndsenii*, *Skeletonema pseudocostatum*, *Skeletonema menzelii*, and *Cyclotella* spp.) were especially important from late spring throughout autumn (Table S2–S6). The mixed and blue states coincided with the decrease of all these diatoms, which were still found in low concentration though, but also of other taxa (i.e., undetermined dinoflagellates and flagellates) with no clear indications of species typical of these conditions. However, possible changes in the community of undetermined small flagellates and naked dinoflagellates would not have been captured by the counts on fixed material. In fact, an abrupt change in the protist community was revealed through molecular methods in the course of a type 2 mixture detected in August 2011^31^.

Both mixture types (Fig. 6c and d) showed diversity values greater than either properly coastal or offshore waters (Fig. 6a and b). This aspect is relevant, since it highlights the role of physical factors in enhancing phytoplankton diversity. The mechanisms behind diversity enhancement could be at least two: either i) advection and mixing of the coastal phytoplankton with species different from the resident ones, or ii) intermediate physical disturbance, which relaxes inter-specific competition by limiting the resource exploitation by fewer dominant species^32,33^. Biological factors uncoupled from physical ones, such as life-cycle transitions, species-specific physiological performances and inter-specific interactions, add further intricacy to phytoplankton community dynamics. In the following sections, we provided some selected examples of temporal changes in phytoplankton communities due to either biological or physical drivers, or to their interplay.

### ‘Autogenic’ transitions within the same phytoplankton community

**Figure 7:**
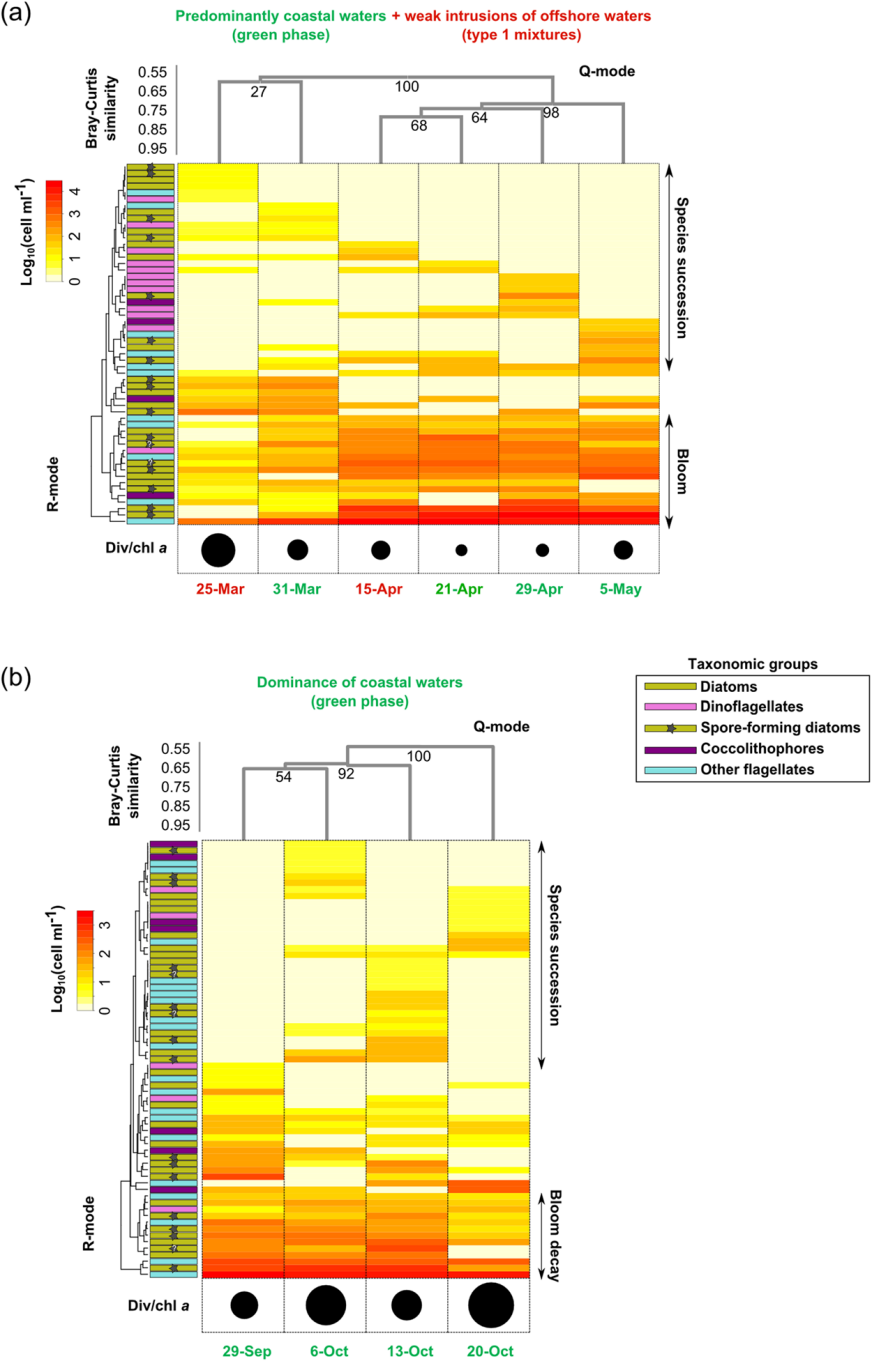
Phytoplankton dynamics during dominant coastal phases in the GoN. **(a)** Spring green (coastal) phase (plus type 1 mixture); **(b)** Autumn green (coastal) phase. Heat maps represent phytoplankton species abundances recorded at LTER-MC during time-periods indicated by dates at the bottom of each panel. The colour of these latter dates (either green, blue or red) indicate the origin of water sampled (either coastal, offshore or mixed, respectively) defined *via* backtracking analysis (see also Fig. 5). Q-mode clustering of phytoplankton data was used to group different phytoplankton samples at consecutive sampling dates; R-mode clustering was used to group species-trends. Numbers at clustering nodes are bootstrap values. Different sized circles below heat maps indicate the ratio between Fisher’s alpha diversity (i.e., Div) and chlorophyll *a* (i.e., chl *a*). Phytoplankton data employed to produce elaborations in panels (**a**) and (**b**) are available in Tables S2–3, respectively.

We first zoomed into phytoplankton dynamics in two cases (Fig. 7) of prevalently coastal functioning of the system (Fig. 6a) despite ephemeral intrusions of offshore waters which induced type 1 mixture conditions (described in Fig. 6c). During an enduring coastal phase, plankton were transported from the coastline to LTER-MC in about 31 h (averagely) (Fig. 4), which is comparable to the time scale needed by most GoN phytoplankton species to complete up to two mitotic divisions during the bloom season^34^. In such a context, and in the absence of intense offshore intrusions, biological processes would be dominant in driving phytoplankton dynamics. Hierarchical clustering analysis (Q- and R-mode) showed changes in the whole phytoplankton community in the investigated period (Fig. 7) with the heat-map particularly highlighting changes in the abundance of individual taxa. In the lowermost scheme of each panel, the scaled black circles showed the change in the ratio between phytoplankton diversity and chl *a*, which characterizes the change in species dominance along the time-period considered.

The first example refers to the time period between March 25 and May 5 (Fig. 7a), a prolonged period during which water to LTER-MC was coming mainly from the coastal sectors (1–2 in Fig. 3a, the green phase in Fig. 6a). R-mode clustering showed two main species-clusters over time: one including species with apparently short-lived blooms (i.e., peaks detected in only one weekly sampling), which showed a succession signal, and a second cluster including species that rose to dominance and formed conspicuous blooms lasting for more than one week (Fig. 7a). The first cluster included mainly dinoflagellates, coccolithophores and other flagellates, while the second included mainly planktonic diatoms (mostly *Skeletonema* and *Thalassiosira* spp.) known to produce resting spores in the coastal waters of GoN^35^. In the presence of persisting coastal waters conditions, the alternation of different species over time can be driven by stochastic biological factors and inter-specific relationships^36^ such as competition, grazing or pathogens. Furthermore, the brisk increase of diatoms after April 15 can be confidently linked to spore germination coupled with increased cell growth rates due to spring conditions, particularly favourable to opportunistic growth in diatoms^4,26^. The rise of diatoms brought an increase in phytoplankton biomass along with a reduction in diversity (black-circles in Fig. 7a). Thus, the variations in the phytoplankton community in the course of a persisting coastal phase of the system can be related to biological processes internal to the community rather than to the impact of external physical drivers (such as the advection of seawater and/or species from offshore).

The second example of a biologically regulated coastal phase refers to the autumnal time period between September 29 and October 20 (Fig. 7b), with water still coming from coastal sectors (1–2 in Fig. 3a, the green phase in Fig. 6a). A large number of species displayed a strong successional signal, while other species, mainly diatoms, underwent evident biomass decay. The most marked of such decays were those of the diatoms *Pseudo-nitzschia* cf. *delicatissima* and *Leptocylindrus* cf. *danicus*, the latter decreased by about 96% in a week. Diatom collapse drove a decrease in the total phytoplankton biomass that was accompanied by a conspicuous increase in diversity (black-circles in Fig. 7b). Vertical transport of plankton particles cannot be invoked as a possible driver of this decrease, since downwelling typically occurs at larger time scales than advection does when the mixed layer exceeds 20 m in depth and the wind blows at a velocity <10 m s^−1^ ^37^, as in the case analysed herein^14,26^. Indeed, an indirect analysis performed in the GoN indicated that at least ~80 h are needed to transport phytoplankton particles along a 20 m deep mixed layer^38^. As for the horizontal scale, the absence of transport of offshore waters towards the coast (as clearly indicated by backtracking, Fig. 5) implies that the changes of species cannot be attributed to physical factors. In the specific context described above, the sudden decay of *L. danicus* can be due to spore production and settlement at the sea bottom, and indeed resting spores of this species are regularly found in the GoN sediments^35^. However, other biological processes such as sinking of the whole population due to physiological changes or selective grazing cannot be ruled out. In the case of *Pseudo-nitzschia* cf. *delicatissima*, a species lacking resting stages, the decay of the bloom can be explained by a massive event of sexual reproduction, like the one that was reported for this species in the autumn of 2006^39^. That sexual event was followed by a brisk decrease in the population, which is consistent with the arrest of vegetative growth following gametogenesis as demonstrated through modelling and laboratory experiments for *Pseudo-nitzschia* species^29,40^.

### ‘Allogenic’ transitions within and between phytoplankton communities

**Figure 8:**
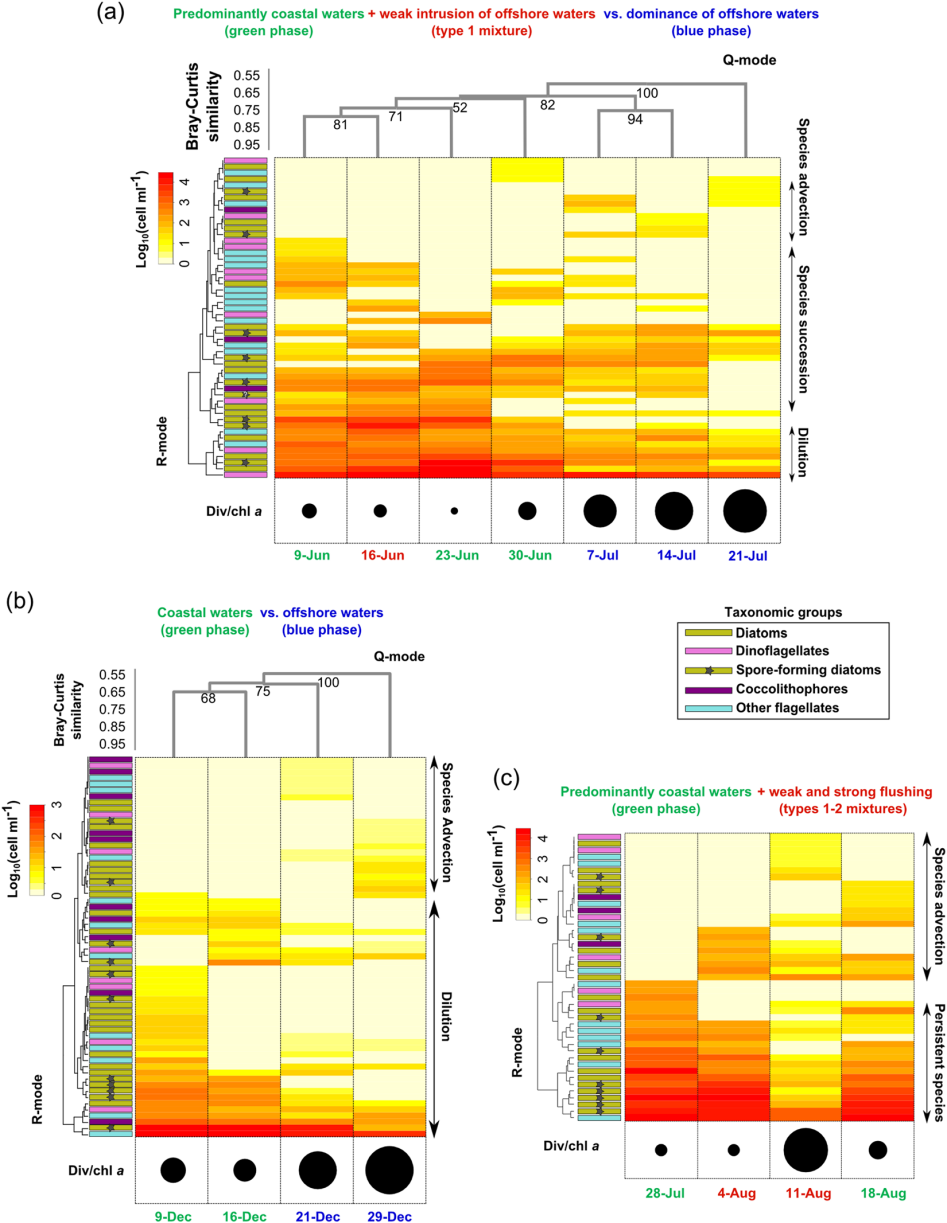
Phytoplankton dynamics during alternations between green and blue phases in the GoN . **(a)** Summer alternation between green and blue phases (plus mixing phase type 1); **(b)** Winter alternation between green and blue phases; **(c)** green phase with type 1–2 mixture. Heat maps represent phytoplankton species abundances recorded at LTER-MC during time-periods indicated by dates at the bottom of each panel. The colour of these latter dates (either green, blue or red) indicate the origin of water sampled (either coastal, offshore or mixed, respectively) defined *via* backtracking analysis (see also Fig. 5). Q-mode clustering of phytoplankton data was used to group different phytoplankton samples at consecutive sampling dates; R-mode clustering was used to group species-trends. Numbers at clustering nodes are bootstrap values. Different sized circles below heat maps indicate the ratio between Fisher’s alpha diversity (i.e., Div) and chlorophyll *a* (i.e., chl *a*). No Q-mode clustering is presented for (**c**) since samples did not group according to time (i.e., 11 August sample was dissimilar from all the other ones). Phytoplankton data employed to produce elaborations in panels (**a**,**b**) and (**c**) are available in Tables S4–6, respectively.

Intense offshore currents occur during the winter and summer months, coming from NE and SW, towards the coast with the maximum speed in the range of 25–30 cm s^−1^ (Fig. 2b to e). These flushing events can advect offshore species and mix different communities. We examined the impact of this flushing on coastal communities by illustrating three cases (Fig. 8), two of which involve waters of closer offshore origin (located in sectors 6–7, Fig. 3a and b), and one offshore waters coming from farther areas of the sectors 5 and 6 (Fig. 3a).

In the first case (June 9–July 21), physical and biological drivers of phytoplankton dynamics coexist (Fig. 8a). During a long-lasting coastal phase (June 9–30, Fig. 5), species succession occurred despite a weak intrusion of offshore waters (type 1 mixture on June 16, Fig. 5, Fig. 6c). On July 7, a flushing event (Fig. 5) involving waters coming from the offshore sector 7 (Fig. 3a) interrupted the coastal phase, causing the transition to the blue offshore phase. This water intrusion led to a drop in the algal biomass and advected different species from offshore, which covered about 20% of the total community (Fig. 8a). Nonetheless, the ongoing succession during the coastal phase continued while an increase in the diversity/chl *a* ratio (black circles, Fig. 8a) was mainly related to the abatement of the most abundant species. The relatively small change in the community composition following the flushing event suggests that, during summer, a wide area of the GoN may be populated by one dominant phytoplankton community. Indeed, the persistent breeze regime determines a 24 h clockwise rotation of the surface current field in the GoN during summer, overall resulting in a relatively high residence time of water masses^14,24,25^. As a consequence, the coastal phytoplankton community, previously transported offshore, may be retained in the outer GoN and eventually be transported back towards the coast, bearing a signal of dilution of the original coastal community.

Unlike in summer, during winter flushing waters even from closer offshore zones (e.g., sector 7, Fig. 3a and b) may have stronger effects on the community structure (Fig. 8b). Surface water circulation in this season oscillates between two opposite scenarios, promoting a sharp alternation between the currents from coast and offshore sectors: SW winds sustain a coastward current field, thus favouring stagnation and retention of surface waters, while NE winds generate an intense coast-offshore jet, permitting a rapid renewal of coastal waters^23,24^. The situation is further complicated because these seasonally defined patterns are occasionally replaced by the formation of cyclonic and anti-cyclonic vortices induced by the Tyrrhenian Sea circulation^23,24^. Following a shift from the coastal to the offshore phase on December 21 (Fig. 5), the abundance of almost all species decreased, while many other species were advected from offshore and mixed with the former (Fig. 8b). On December 29, the meridional spreading (Supplementary Note) of VPPs was much higher than on previous dates. Unlike in summer, when a single community dominated the GoN, the shift between the green and blue phases during winter can mix at least two different communities with distinct features, one coastal and one offshore, the first showing higher biomass and lower diversity and the second showing the opposite features (black circles in Fig. 8b).

In some cases, phytoplankton community modifications induced by physical factors can be even more conspicuous than those described above. This happens under strong flushing events, when the coastal area is ‘invaded’ by the arrival of offshore waters originating not only from the nearest (sector 7) but also from the outer sectors of the GoN (sectors 5–6, Fig. 3a). Specifically, a relatively long coastal phase between July 28 and August 18 was interrupted by offshore water inputs on August 4 and 11 (Fig. 5). On the former date, offshore waters came from the nearest sector 7 causing a small change in the community composition and virtually no change in the ecological features like phytoplankton biomass and diversity (black circles, Fig. 8c). Apparently, the offshore intrusion occurring on August 4 only involved the re-transportation of an ‘older’, diluted coastal water towards the coast, similarly to what occurred in July (Fig. 8a). Conversely, flushing on August 11 determined a decrease in phytoplankton biomass due to strong dilution and an increase in diversity (black circles in Fig. 8c): about half of the species detected during the above-mentioned time period were advected from the relatively far offshore sectors. A study based on high throughput sequencing and metabarcoding of protist communities revealed a similar case of sudden and complete replacement of the coastal community in August 2011, which was also interpreted as the consequence of offshore water intrusion from the farthest sector of the GoN^31^. After these sudden changes, the community can however revert to a coastal phase that is more similar to the one preceding the flushing^31^, as also observed on August 18 in this study.

### Concluding remarks

The approach adopted in this study allowed disentangling the respective roles of physical and biological forcings in driving phytoplankton dynamics in the GoN and lead to the following synthesis.

Biological, ‘autogenic’ factors, such as production/germination of resting stages, species-specific physiological performances and interspecific-interactions, all inducing species successions, are determinant drivers of modifications in coastal phytoplankton communities in the course of long-lasting green phases. Conversely, physical, ‘allogenic’ factors are determinant in driving coastal phytoplankton, *via* dilution and species advection, when blue waters either i) substitute green waters or ii) invade green waters for shorter time periods. In the latter cases, the farther the origin zone of offshore waters, the larger the modification induced in the phytoplankton community. Between these extremes, both biological and physical drivers interplay in cases of less intense horizontal mixing, involving waters originating from the offshore origin zone closer to the coast.

The alternation between coastal and offshore water masses promote phytoplankton diversity, because the dilution in the phytoplankton density may decrease the impact of the dominating species over the available resources, which is enhanced during prolonged coastal phases. This ‘intermediate disturbance’ mechanism often explains diversity oscillations in plankton communities^32,33^ and we showed clear cases of this coupled physical-biological dynamics.

The above insights on the relative contributions of water currents and species’ ecology–autoecology in the shaping of coastal phytoplankton communities were possible only after complementing the hydrographic and ecological information. Beyond the study site, our approach represents a proof-of-concept that can be applied at a more general level to investigate the processes leading to the establishment of specific coastal plankton communities, while at the same time it sheds light on processes which modulate the fluxes from the land into the open sea. Ultimately, such efforts may be useful in supporting decisions and solving problems in coastal management or in the study of harmful algal blooms.

## Methods

The synoptic, basin-scale, near real-time surface circulation of the GoN was monitored through a network of coastal HFRs (SeaSonde, CODAR Ocean Systems, Mountain View, USA) since October 2004. An exhaustive technical description of the functioning of the HFR systems is available in previous publications^11,41^. The HF radar network in the GoN has been employed over the years to study both the surface currents^10,14,23–25,42,43^ and the wave field^43^. The current fields used in the present study were acquired using three transceiving antennae working at 25 MHz (Fig. 1), a configuration operating since May 2008 ensuring an optimal coverage of the basin, with a spatial resolution of 1.0 × 1.0 km^14,23,43^. Owing to the working frequency, the measured currents refer to a depth of 0.5–1.0 m from the surface^44^. As HFR derived currents may be affected by different sources of error^45^, antenna pattern measurements were conducted routinely to correct currents measured in the GoN returning precise and accurate estimates of the surface fields^9,46^. Our analysis is focused on 2009; this year was selected as it presented a minimal number of gaps in terms of radar data density and associated weekly biological sampling.

### Lagrangian backward trajectories

The backward simulations were developed by applying an Eulerian/Lagrangian model (GNOME- General NOAA Operational Modeling Environment)^47^ that was conceived as a multi-purpose trajectory model to estimate passive particle trajectories by processing oceanographic as well as atmospheric data for a fixed geographic region^48,49^. In our study, the surface velocity fields detected by the HFR system between January 1 and December 31, 2009 provided the advective component of the Lagrangian model. The backward mode is indeed computationally advantageous to this extent when the number of receptors of VPPs is less than the number of sources considered^15^, as in our study site. The backtracking simulations were run considering the HFR currents in reverse time and direction by applying a 10 min time-step integration. The accuracy of the GoN HFR system was recently validated by comparison with surface drifter data^9,46^; however, in order to account for uncertainties in the current data, a ±15% error estimate was set in the Lagrangian model, in both along- and cross-current directions, in line with previous literature^50–52^. No horizontal diffusion was considered, the irreversible nature of the random processes causing a negative diffusion equivalent to a physically inconsistent aggregation in the backward mode. For all the weekly dates monitored at LTER-MC during 2009 (49 weeks), we simulated backward-in-time trajectories by releasing VPPs of 10,000 independent particles on a 1.0 × 1.0 km grid centred on the LTER-MC site. By means of the backward trajectories, the VPP location up to 4 days (i.e., 96 h) prior to its arrival at LTER-MC was estimated. The positions of the VPPs at the end of each backward simulation were used to evaluate the origin zones of phytoplankton organisms, distinguishing periods during which VPPs originated from the inner part of the basin (coastal phases) or from the open waters (offshore phases).

To define the spatial distribution of the plankton origin zones, a binned cumulative probability *p*_*bin*_ was calculated. The entire basin was divided into regular subsectors (bins) (0.01° longitude × 0.01° latitude, approximately 1 km^2^) and, for each weekly simulation, the final backward position of a subset of 1,000 particles was analysed. The total number of particles originating from the same bin (*N*_*bin*_) was cumulated over the entire yearly set of model runs (49 weeks). For each particle, the relative percentage contribution was calculated as *p*_*i*_ = 1/49,000. Subsequently, for each bin, the binned cumulative probability *p*_*bin*_ was computed as follows:

1 $${p}_{{\rm{bin}}}=\sum _{i=1}^{{N}_{bin}}{p}_{i}$$

To interpret changes in the community composition among samples and during possible transitions between offshore and coastal phases, we computed the difference between the VPPs release time at LTER-MC and the first-entry time (T_A_) of VPPs in the origin zones. In particular, we computed the T_A_ of the particles in an area neighbouring the VPPs centre mass (C_m_ (*x*_*c*_, *y*_*c*_)), reinterpreting the receptor-based method discussed elsewhere^53^. For each simulation, the neighbourhood area around C_m_ was defined as the region enclosing the 1/20^th^ closest particles by the end of the run. For each particle inside this area, the projected distances from C_m_ (d_x_ and d_y_, respectively) were calculated, along with their overall standard deviations (σ_d_x_ and σ_d_y_). The projected bandwidths of such neighbouring area centred on C_m_ (L_x_ and L_y_, respectively) were then taken as follows:

2a $${{\rm{L}}}_{{\rm{x}}}:\,{\rm{\min }}\{{\rm{\max }}({{\rm{d}}}_{{\rm{x}}}),2{\rm{\sigma }}\_{{\rm{d}}}_{{\rm{x}}}\}$$

2b $${{\rm{L}}}_{{\rm{y}}}:\,{\rm{\min }}\{{\rm{\max }}({{\rm{d}}}_{{\rm{y}}}),2{\rm{\sigma }}\_{{\rm{d}}}_{{\rm{y}}}\}$$

The first-entry time of particles accessing this neighbouring area and remaining inside it until the end of the simulation was then calculated and used to estimate the minimum and average T_A_ for each VPP.

### Integration between physical and ecological observations

The final backward position of the VPPs was compared with the physical and chemical characteristics of the corresponding LTER-MC samples based on salinity and chl *a* values, following previous observation by some authors of the present paper^30^. These analyses were performed separately for the winter (December, January and February), spring (March, April and May), summer (June, July and August), and autumn (September, October and November) samples.

The zonal and meridional dispersion (*S*_*xx*_ and *S*_*yy*_) around the mean particle displacement of each VPP was also computed^54^ (Supplementary Note), as follows:

3 $$\begin{array}{cc}{S}_{xx}=\langle {(x-\langle x\rangle )}^{2}\rangle  & {S}_{yy}=\langle {(y-\langle y\rangle )}^{2}\rangle \end{array}$$

The index of spreading *Spr* is the sum of the zonal and the meridional dispersion:

4 $$Spr={S}_{xx}+{S}_{yy}$$

The index of spreading *(Spr)*, the first-entry time (T_A_), salinity, chl *a* and phytoplankton diversity (Fisher’s alpha index of diversity, see also below) for each analyzed date were compared by standardizing each dataset, with the following formula:

$$y\,=\frac{(x-mean\,(x))}{std\,(x)}$$

in which x is the source variable (e.g., salinity), y is the standardized variable and *std* is the standard deviation of x. All these datasets were aggregated based on the relative similarity between dates and the median value for each variable was taken as a reference for the main groups of sampling dates.

The physical-chemical-biological analyses of plankton water samples from LTER-MC were performed following^26^. Chlorophyll *a* was estimated by means of direct extraction from water samples^26^. The ecological characterization of the water masses arriving at LTER-MC was obtained by means of cluster analyses based on salinity and chl *a* values (according to^30^). Q-mode clustering was based on Bray-Curtis similarity^55^, and calculated over log-transformed abundance data. The Fisher’s alpha index of diversity^56^ was used to parameterize biological diversity in phytoplankton samples. This index is theoretically independent of sample size^57^, and is considered as a robust diversity discriminator for samples featuring a small fraction of abundant species and a large proportion of ‘rare’ species when abundance of species follows a log-series distribution^58^, as in our dataset. Statistical analyses were performed with the software PAST ver. 1.89^59^ and R^60^.

### Data availability

The 2009 HFR data (in NetCDF format) are in the process of being included into the Flagship Project RITMARE radar public data bank. The HF data for year 2009 are presently available on request to the corresponding author. The biological dataset supporting this article have been uploaded as part of the Supplementary Information.

## Electronic supplementary material

Supplementary material

## References

[1] Reynolds CS (1984). Phytoplankton periodicity: the interactions of form, function and environmental variability. Freshwater Biology.

[2] Smayda, T. In *The Physiological Ecology of Phytoplankton* (ed I. Morris) 493–570 (Blackwell Scientific Publications, 1980).

[3] Wyatt T, Zingone A (2014). Population dynamics of red tide dinoflagellates. Deep Sea Research Part II: Topical Studies in Oceanography.

[4] Wyatt T (2014). Margalef’s mandala and phytoplankton bloom strategies. Deep Sea Research II.

[5] Schapira M, Vincent D, Gentilhomme V, Seuront L (2008). Temporal patterns of phytoplankton assemblages, size spectra and diversity during the wane of a *Phaeocystis globosa* spring bloom in hydrologically contrasted coastal waters. Journal of the Marine Biological Association of the United Kingdom.

[6] Berdalet E (2016). Marine harmful algal blooms, human health and wellbeing: challenges and opportunities in the 21st century. Journal of the Marine Biological Association of the United Kingdom.

[7] Cloern JE, Jassby AD (2010). Patterns and scales of phytoplankton variability in estuarine-coastal ecosystems. Estuaries and Coasts.

[8] Martin AP, Zubkov MV, Burkill PH, Holland RJ (2005). Extreme spatial variability in marine picoplankton and its consequences for interpreting Eulerian time-series. Biology Letters.

[9] Bellomo L (2015). Toward an integrated HF radar network in the Mediterranean Sea to improve search and rescue and oil spill response: the TOSCA project experience. Journal of Operational Oceanography.

[10] Cianelli, D. *et al*. In *Mediterranean Ecosystems: Dynamics, Management and Conservation* (ed G. S. Williams) 129–150 (Nova Science Publishers, Inc., 2012).

[11] Paduan JD, Washburn L (2013). High-frequency radar observations of ocean surface currents. Annual Review of Marine Science.

[12] Abascal AJ, Castanedo S, Medina R, Losada IJ, Alvarez-Fanjul E (2009). Application of HF radar currents to oil spill modelling. Marine Pollution Bulletin.

[13] Abascal AJ, Castanedo S, Fernández V, Medina R (2012). Backtracking drifting objects using surface currents from high-frequency (HF) radar technology. Ocean Dynamics.

[14] Uttieri M (2011). Multiplatform observation of the surface circulation in the Gulf of Naples (Southern Tyrrhenian Sea). Ocean Dynamics.

[15] Seibert P, Frank A (2004). Source-receptor matrix calculation with a Lagrangian particle dispersion model in backward mode. Atmospheric Chemistry and Physics.

[16] Koracin D (2011). Regional source identification using Lagrangian stochastic particle dispersion and HYSPLIT backward-trajectory models. Journal of the Air & Waste Management Association.

[17] Batchelder HP (2006). Forward-in-Time-/Backward-in-Time-Trajectory (FITT/BITT) modeling of particles and organisms in the coastal ocean. Journal of Atmospheric and Oceanic Technology.

[18] Iermano I, Moore AM, Zambianchi E (2016). Impacts of a 4-dimensional variational data assimilation in a coastal ocean model of southern Tyrrhenian Sea. Journal of Marine Systems.

[19] Falco P, Trani M, Zambianchi E (2016). Water mass structure and deep mixing processes in the Tyrrhenian Sea: Results from the VECTOR project. Deep Sea Research Part I: Oceanographic Research Papers.

[20] Carrada GC (1980). Variability in the hydrographic and biological features of the Gulf of Naples. P. S. Z. N. I. Marine Ecology.

[21] Marino, D., Modigh, M. & Zingone, A. In *Marine Phytoplankton Productivity*. *Lecture Notes on Coastal and Estuarine Studies* (eds O. Holm-Hansen, L. Bolis, & R. Gilles) 89–100 (Springer, 1984).

[22] Zingone A, Montresor M, Marino D (1990). Summer phytoplankton physiognomy in coastal waters of the Gulf of Naples. P. S. Z. N. I. Marine Ecology.

[23] Cianelli D (2015). Inshore/offshore water exchange in the Gulf of Naples. Journal of Marine Systems.

[24] Cianelli, D. *et al*. In *Remote Sensing: Techniques, Applications and* Technologies (ed E. Alcântara) 1–30 (Nova Science Publishers, Inc., 2013).

[25] Menna M, Mercatini A, Uttieri M, Buonocore B, Zambianchi E (2007). Wintertime transport processes in the Gulf of Naples investigated by HF radar measurements of surface currents. Il Nuovo Cimento.

[26] Ribera d’Alcalà M (2004). Seasonal patterns in plankton communities in a pluriannual time series at a coastal Mediterranean site (Gulf of Naples): an attempt to discern recurrences and trends. Scientia Marina.

[27] Zingone A (2010). Coastal phytoplankton do not rest in winter. Estuaries and Coasts.

[28] D’Alelio D, Libralato S, Wyatt T, Ribera d’Alcalà M (2016). Ecological-network models link diversity, structure and function in the plankton food-web. Scientific Reports.

[29] D’Alelio D (2010). The time for sex: a biennial life cycle in a marine planktonic diatom. Limnology and Oceanography.

[30] D’Alelio D (2015). The green-blue swing: plasticity of plankton food-webs in response to coastal oceanographic dynamics. Marine Ecology.

[31] Piredda R (2017). Diversity and temporal patterns of planktonic protist assemblages at a Mediterranean Long Term Ecological Research site. FEMS Microbiology Ecology.

[32] Huston, M. A general hypothesis of species diversity. *The American Naturalist* **113**, 81–101, www.jstor.org/stable/2459944 (1979).

[33] Reynolds CS, Padisák J, Sommer U (1993). Intermediate disturbance in the ecology of phytoplankton and the maintenance of species diversity: a synthesis. Hydrobiologia.

[34] D’Alelio D (2016). Plankton food-webs: to what extent can they be simplified?. Advances in Limnology and Oceanography.

[35] Montresor M, Di Prisco C, Sarno D, Margiotta F, Zingone A (2013). Diversity and germination patterns of diatom resting stages at a coastal Mediterranean site. Marine Ecology Progress Series.

[36] Scheffer M, Rinaldi S, Huisman J, Weissing FJ (2003). Why plankton communities have no equilibrium: solutions to the paradox. Hydrobiologia.

[37] Denman KL, Gargett AE (1983). Time and space scales of vertical mixing and advection of phytoplankton in the upper ocean. Limnology and Oceanography.

[38] Brunet C, Casotti R, Aronne B, Vantrepotte V (2003). Measured photophysiological parameters used as tools to estimate vertical water movements in the coastal Mediterranean. Journal of Plankton Research.

[39] Sarno D, Zingone A, Montresor M (2010). A massive and simultaneous sex event of two *Pseudo-nitzschia* species. Deep Sea Research II.

[40] Scalco E, Stec K, Iudicone D, Ferrante MI, Montresor M (2014). The dynamics of sexual phase in the marine diatom *Pseudo-nitzschia multistriata* (Bacillariophyceae). Journal of Phycology.

[41] Paduan JD, Graber HC (1997). Introduction to High-Frequency radar: reality and myth. Oceanography.

[42] Serafino F (2012). REMOCEAN: a flexible X-band radar system for sea-state monitoring and surface current estimation. IEEE Geoscience and Remote Sensing Letters.

[43] Falco P (2016). Dynamics and sea state in the Gulf of Naples: potential use of high-frequency radar data in an operational oceanographic context. Journal of Operational Oceanography.

[44] Stewart RH, Joy JW (1974). HF radio measurements of surface currents. Deep Sea Research.

[45] Laws K, Paduan JD, Vesecky J (2010). Estimation and assessment of errors related to antenna pattern distortion in CODAR SeaSonde high-frequency radar ocean current measurements. Journal of Atmospheric and Oceanic Technology.

[46] Kalampokis A, Uttieri M, Poulain P-M, Zambianchi E (2016). Validation of HF radar-derived currents in the Gulf of Naples with Lagrangian data. IEEE Geoscience and Remote Sensing Letters.

[47] Zelenke, B., O’Connor, C., Barker, C. J., Beegle-Krause, C. J. & Eclipse, L. *General NOAA Operational Modeling Environment (GNOME) Technical Documentation. Dept. of Commerce, NOAA Technical Memorandum NOS OR&R 40*. (Emergency Response Division, NOAA, 2012).

[48] Beegle-Krause, C. J. In *IOSC**2001**Proceedings**V*ol. 2 865-871 (Mira Digital Publishing, Inc., 2001).

[49] Engie K, Klinger T (2007). Modeling passive dispersal through a large estuarine system to evaluate marine reserve network connections. Estuaries and Coasts.

[50] Chapman RD (1997). On the accuracy of HF radar surface current measurements: intercomparisons with ship-based sensors. Journal of Geophysical Research.

[51] Emery BM, Washburn L, Harlan JA (2004). Evaluating radial current measurements from CODAR high-frequency radars with moored current meters. Journal of Atmospheric and Oceanic Technology.

[52] Paduan JD, Kim KC, Cook MS, Chavez FP (2006). Calibration and validation of direction-finding high-frequency radar ocean surface current observations. IEEE Journal of Oceanic Engineering.

[53] Vitali L (2006). Validation of a Lagrangian dispersion model implementing different kernel methods for density reconstruction. Atmospheric Environment.

[54] Zambianchi E, Griffa A (1994). Effects of finite scales of turbulence on dispersion estimates. Journal of Marine Research.

[55] Bray JR, Curtis JT (1957). An ordination of the upland forest communities of Southern Wisconsin. Ecological Monographs.

[56] Fisher RA, Corbet AS, Williams CB (1943). The relation between the number of species and the number of individuals in a random sample of an animal population. Journal of Animal Ecology.

[57] Kempton RA (1979). The structure of species abundance and measurement of diversity. Biometrics.

[58] Taylor LR, Kempton RA, Woiwod IP (1976). Diversity statistics and the log-series model. Journal of Animal Ecology.

[59] Hammer Ø, Harper DAT, Ryan PD (2001). PAST: paleontological statistics software package for education and data analysis. Palaeontologia Electronica.

[60] R Core Team. *R: a Language and Environment for Statistical Computing*. (R Foundation for Statistical Computing, 2013).

