# Supplementary material for: Disentangling physical and biological drivers of phytoplankton dynamics in a coastal system
Source: Sci Rep. 2017 Nov 20;7:15868. doi: 10.1038/s41598-017-15880-x (PMC5696475; doi:10.1038/s41598-017-15880-x)
Supplement: Supplementary file 1 — Supplementary material [file 41598_2017_15880_MOESM1_ESM.pdf]

Disentangling physical and biological drivers of phytoplankton dynamics in a coastal system

Cianelli D.[1,2, 3, *], D'Alelio D. [4, *], Uttieri M. [1,3,4], Sarno D. [4], Zingone A. [4], Zambianchi E. [1,3,5] , Ribera d'Alcalà M. [4]

(1) Dipartimento di Scienze e Tecnologie, Università degli Studi di Napoli "Parthenope", Centro Direzionale di Napoli – Isola C4, 80143 Naples, Italy
(2) ISPRA – Istituto Superiore per la Protezione e la Ricerca Ambientale, Via Vitaliano Brancati 60 - 00144 Rome, Italy
(3) CoNISMa (Consorzio Nazionale Interuniversitario per le Scienze del Mare), Piazzale Flaminio 9, 00196 Rome, Italy
(4) Stazione Zoologica Anton Dohrn, Villa Comunale, 80121 Naples, Italy
(5) ISAC-CNR, Via Fosso del Cavaliere 100, 00133 Rome, Italy
* These authors contributed equally to this work

Supplementary Info:

- *Table S1. Data of salinity and chlorophyll a collected at station LTER-MC during 2009.*
- *Tables S2. Data of phytoplankton abundance, biomass, diversity plus chl a from station LTER-MC and used to produce elaborations in Fig. 7a.*
- *Tables S3. Data of phytoplankton abundance, biomass, diversity plus chl a from station LTER-MC and used to produce elaborations in Fig. 7b.*
- *Tables S4. Data of phytoplankton abundance, biomass, diversity plus chl a from station LTER-MC and used to produce elaborations in Fig. 8a.*
- *Tables S5. Data of phytoplankton abundance, biomass, diversity plus chl a from station LTER-MC and used to produce elaborations in Fig. 8b.*
- *Tables S6. Data of phytoplankton abundance, biomass, diversity plus chl a from station LTER-MC and used to produce elaborations in Fig. 8c.*
- *Supplementary Note: VPPs zonal and meridional spreading.*

Supplementary Tables

Table S1. Data of salinity and chlorophyll *a* collected at station LTER-MC during 2009.

	Average value (depth 0-2 m)	
**Date**	**Salinity**	**Chlorophyll a (µg L^{-1})**
7.1.09	38.03	0.67
13.1.09	38.05	0.53
22.1.09	36.99	1.73
29.1.09	37.18	1.77
13.2.09	37.84	1.02
17.2.09	37.91	0.71
24.2.09	37.85	0.95
3.3.09	37.40	4.70
10.3.09	37.24	7.53
17.3.09	37.77	1.55
25.3.09	37.87	1.83
31.3.09	37.33	4.37
15.4.09	37.57	3.32
21.4.09	37.38	7.40
29.4.09	37.22	6.85
5.5.09	not available	2.28
12.5.09	37.25	5.02
26.5.09	37.07	6.55
3.6.09	36.63	6.74
9.6.09	36.90	5.64
16.6.09	37.08	5.69
23.6.09	36.83	13.51
30.6.09	37.12	3.40
7.7.09	37.46	1.08
14.7.09	37.56	0.85
21.7.09	37.64	0.49
28.7.09	37.35	4.44
4.8.09	37.03	5.31
11.8.09	37.62	0.78
18.8.09	37.35	3.01
25.8.09	37.42	4.91
1.9.09	37.87	0.31
8.9.09	38.00	0.36
16.9.09	37.63	2.14
22.9.09	37.79	0.99
29.9.09	37.68	2.37
6.10.09	37.90	1.41
13.10.09	37.70	2.97
20.10.09	37.91	1.00
27.10.09	37.81	1.55
4.11.09	37.82	1.67
11.11.09	37.18	1.44
17.11.09	37.43	6.20
24.11.09	37.70	2.79
2.12.09	37.28	4.39
9.12.09	37.52	3.09
16.12.09	37.71	2.31
21.12.09	37.80	1.24
29.12.09	37.75	0.86

Tables S2. Data of phytoplankton abundance, biomass, diversity and chlorophyll *a* from station LTER-MC and used to produce elaborations in Fig. 7a.

		Date					
		**25.3.09**	**31.3.09**	**14.4.09**	**21.4.09**	**29.4.09**	**5.5.09**
**Taxonomic group**	**Species name**	**Concentration (cell mL^{-1}, depth 0 m)**					
Diatom	*Asterionellopsis glacialis*	79	255	0	0	0	0
Diatom	*Bacteriastrum parallelum*	0	33	177	66	186	297
Diatom	Centric diatoms < 5 µm	3	233	177	399	520	37
Diatom	*Cerataulina pelagica*	10	11	89	0	0	0
Diatom	*Chaetoceros affinis*	10	0	0	0	0	0
Diatom	*Chaetoceros anastomosans*	10	0	0	0	0	0
Diatom	*Chaetoceros contortus*	511	177	0	0	112	0
Diatom	*Chaetoceros curvi-curv*i	0	44	244	709	112	223
Diatom	*Chaetoceros curvisetus*	36	133	0	0	0	0
Diatom	*Chaetoceros decipiens*	0	22	0	0	0	0
Diatom	*Chaetoceros diadema*	7	22	0	0	0	0
Diatom	*Chaetoceros diversus*	0	0	0	0	186	0
Diatom	*Chaetoceros socialis*	0	11	66	66	0	260
Diatom	*Chaetoceros* spp.	49	222	377	421	409	1041
Diatom	*Chaetoceros tenuissimus*	7	78	687	953	372	520
Diatom	*Chaetoceros wighamii*	0	0	0	0	0	112
Diatom	*Cylindrotheca closterium*	0	11	0	0	0	74
Diatom	*Dactyliosolen blavyanus*	0	0	22	0	0	0
Diatom	*Leptocylindrus danicus*	3	44	66	266	223	0
Diatom	*Lioloma* spp.	0	11	0	0	0	0
Diatom	Pennate diatoms < 10 µm	7	0	0	0	0	0
Diatom	*Proboscia alata*	3	11	0	0	0	0
Diatom	*Pseudo-nitzschia delicatissima*	46	155	66	0	0	186
Diatom	*Pseudo-nitzschia galaxiae*	13	0	465	310	149	1449
Diatom	*Pseudo-nitzschia galaxiae* "small morphotype"	20	66	44	44	37	0
Diatom	*Pseudo-nitzschia pseudodelicatissima*	16	66	0	0	0	0
Diatom	*Skeletonema pseudocostatum*	0	55	1573	7334	9923	15759
Diatom	*Thalassionema bacillare/frauenfeldii*	10	0	0	0	0	0
Diatom	*Thalassiosira* spp.	0	11	2925	1485	2564	669
Dinoflagellate	*Heterocapsa minima*	0	0	0	0	37	0
Dinoflagellate	*Heterocapsa niei*	0	0	0	0	0	37
Dinoflagellate	*Lessardia elongata*	0	0	0	0	37	0
Dinoflagellate	*Mesoporos adriaticus*	3	11	0	0	0	0
Dinoflagellate	Naked dinoflagellates < 15 µm	20	44	199	465	372	223
Dinoflagellate	Naked dinoflagellates > 15 µm	0	0	22	66	37	0
Dinoflagellate	Naked dinoflagellates > 30 µm	0	0	44	0	0	0
Dinoflagellate	*Oxytoxum variabile*	0	0	0	0	37	0
Dinoflagellate	*Prorocentrum triestinum*	0	0	0	22	74	0
Dinoflagellate	*Protoperidinium* spp.	0	0	0	22	0	0
Dinoflagellate	Thecate dinoflagellates < 15 µm	10	0	22	44	0	0
Dinoflagellate	Thecate dinoflagellates < 15 µm sp.1	3	0	0	0	0	0

Coccolithophore	*Emiliania huxleyi*	43	100	0	66	0	37

Table S2 (cont'd)

Coccolithophore	*Sphaerocalyptra quadridentata*	0	0	0	0	0	37
Coccolithophore	*Syracosphaera pulchra*	0	11	0	0	37	0
Coccolithophore	Undetermined coccolithophores	10	11	22	0	74	112
Other flagellate	*Diplostauron* cf. *elegans*	0	11	0	0	0	0
Other flagellate	*Eutreptiella* spp.	3	0	0	0	0	0
Other flagellate	Heterotroph flagellates	33	11	266	0	2007	112
Other flagellate	*Leucocryptos marina*	3	0	22	66	37	74
Other flagellate	*Ollicola vangoorii*	3	89	66	44	37	149
Other flagellate	*Paulinella ovalis*	0	22	66	66	74	37
Other flagellate	*Pseudoscourfieldia marina*	0	0	22	22	0	74
Other flagellate	*Pyramimonas* spp.	0	22	0	66	74	74
Other flagellate	*Tetraselmis* spp.	0	0	0	0	0	74
Other flagellate	Undetermined cryptophyceans < 10 µm	26	89	332	377	260	409
Other flagellate	Undetermined phytoflagellates < 10 µm	619	3002	8309	10901	7210	7805
	**Phytoplankton group**	**Biomass (µg C L^{-1}, depth 0 m)**					
	Diatoms	59.0	64.9	203.8	253.7	320.8	381.4
	Dinoflagellates	3.0	4.9	135.9	122.5	75.9	22.8
	Coccolithophores	1.3	4.8	0.7	1.5	9.9	6.8
	Other flagellates	4.3	17.8	50.2	62.9	61.7	52.4
	Total phytoplankton	67.6	92.4	390.6	440.6	468.3	463.4
		**Diversity - Fisher's alpha**					
	Total phytoplankton	5.2	4.7	3.0	2.6	3.0	2.8
		**Chlorophyll *a* (µg L^{-1}, 0-2 m, average value)**					
		1.83	4.37	3.32	7.40	6.85	2.28

Tables S3. Data of phytoplankton abundance, biomass, diversity plus chl *a* from station LTER-MC and used to produce elaborations in Fig. 7b.

		Date			
		**29.9.09**	**6.10.09**	**13.10.09**	**20.10.09**
**Taxonomic group**	**Species name**	**Concentration (cell mL^{-1}, depth 0 m)**			
Diatom	Centric diatoms < 5 µm	79	0	46	7
Diatom	*Cerataulina pelagica*	33	0	10	7
Diatom	*Chaetoceros anastomosans*	0	10	0	0
Diatom	*Chaetoceros curvi-curvi*	0	13	0	0
Diatom	*Chaetoceros curvisetus*	461	0	10	0
Diatom	*Chaetoceros rostratus*	0	0	7	0
Diatom	*Chaetoceros simplex*	0	0	3	0
Diatom	*Chaetoceros socialis*	59	16	3	0
Diatom	*Chaetoceros* spp.	119	138	89	20
Diatom	*Chaetoceros tenuissimus*	26	49	26	13
Diatom	*Cyclotella atomus* var. *gracilis*	0	16	33	0
Diatom	*Cyclotella* spp.	0	0	0	20
Diatom	*Cylindrotheca closterium*	0	10	0	3
Diatom	*Dactyliosolen blavyanus*	0	0	3	0
Diatom	*Dactyliosolen fragilissimus*	7	0	0	0
Diatom	*Dactyliosolen phuketensis*	7	0	0	3
Diatom	*Lauderia annulata*	33	7	10	13
Diatom	*Leptocylindrus danicus*	560	820	906	40
Diatom	*Leptocylindrus mediterraneus*	0	0	3	0
Diatom	*Leptocylindrus minimus*	59	3	76	0
Diatom	*Lioloma* spp.	0	0	0	3
Diatom	Pennate diatoms < 10 µm	0	0	0	3
Diatom	Pennate diatoms > 10 µm	0	3	10	0
Diatom	*Pseudo-nitzschia delicatissima*	112	155	227	40
Diatom	*Pseudo-nitzschia galaxiae*	7	0	10	0
Diatom	*Pseudo-nitzschia multistriata*	0	3	3	26
Diatom	*Pseudo-nitzschia pseudodelicatissima*	171	102	119	0
Diatom	*Skeletonema menzelii*	13	16	99	10
Diatom	*Skeletonema pseudocostatum*	0	49	30	0
Diatom	*Skeletonema tropicum*	0	0	13	0
Diatom	*Thalassionema nitzschioides*	0	10	10	7
Diatom	*Thalassiosira* cf. *allenii*	0	0	23	0
Diatom	*Thalassiosira oestrupii* var. *venrickae*	0	3	0	0
Diatom	*Thalassiosira* spp.	105	102	56	10
Diatom	*Thalassiosira tealata*	79	23	336	0
Dinoflagellate	*Mesoporos adriaticus*	7	0	0	0
Dinoflagellate	Naked dinoflagellates < 15 µm	7	33	23	30
Dinoflagellate	Naked dinoflagellates > 15 µm	7	0	7	0
Dinoflagellate	*Oxytoxum variabile*	0	0	0	3
Dinoflagellate	Thecate dinoflagellates < 15 µm	0	3	0	3

Coccolithophore	*Acanthoica quattrospina*	0	0	0	3
Coccolithophore	*Algirosphaera robusta*	0	3	0	0
Coccolithophore	*Calciopappus caudatus*	66	26	0	0

Table S3 (cont'd)

Coccolithophore	*Calciosolenia murrayi*	0	0	0	3
Coccolithophore	*Emiliania huxleyi*	33	20	0	214
Coccolithophore	*Syracosphaera pulchra*	0	3	0	0
Coccolithophore	Undetermined coccolithophores	26	10	0	13
Other flagellate	Coccoids	455	267	201	201
Other flagellate	*Hemiselmis* spp.	53	0	0	0
Other flagellate	Heterotroph flagellates	0	0	49	280
Other flagellate	*Leucocryptos marina*	0	0	16	0
Other flagellate	*Ollicola vangoorii*	13	16	16	10
Other flagellate	*Pachysphaera* spp.	0	3	0	0
Other flagellate	*Paraphysomonas* spp.	0	0	16	0
Other flagellate	*Paulinella ovalis*	7	7	3	0
Other flagellate	*Phaeocystis* spp.	0	0	0	30
Other flagellate	*Plagioselmis* spp.	0	0	10	0
Other flagellate	*Pseudoscourfieldia marina*	33	13	10	3
Other flagellate	*Pyramimonas* spp.	7	0	10	7
Other flagellate	*Rhizomonas setigera*	0	0	33	0
Other flagellate	*Tetraselmis* spp.	0	0	3	0
Other flagellate	Undetermined cryptophyceans < 10 µm	119	63	59	13
Other flagellate	Undetermined cryptophyceans > 10 µm	0	3	0	0
Other flagellate	Undetermined phytoflagellates < 10 µm	2800	1189	1080	1034
Other flagellate	Undetermined phytoflagellates > 10 µm	0	3	7	0
Other flagellate	Undetermined prasinophyceae	7	0	0	0
Other flagellate	Undetermined prymnesiophyceae	0	0	3	0
	**Phytoplankton group**	**Biomass (µg C L^{-1}, depth 0 m)**			
	Diatoms	108.7	70.0	93.8	14.7
	Dinoflagellates	6.9	2.6	7.0	2.5
	Coccolithophores	2.4	1.9	0.0	5.4
	Other flagellates	17.3	8.4	9.7	8.7
	Total phytoplankton	135.2	82.9	110.6	31.2
		**Diversity - Fisher's alpha**			
	Total phytoplankton	4.5	5.7	6.8	5.2
		**Chlorophyll *a* (µg L^{-1}, 0-2 m, average value)**			
		2.37	1.41	2.97	1.00

Tables S4. Data of phytoplankton abundance, biomass, diversity plus chl *a* from station LTER-MC and used to produce elaborations in Fig. 8a.

		Date						
		**9.6.09**	**16.6.09**	**23.6.09**	**30.6.09**	**7.7.09**	**14.7.09**	**21.7.09**
**Taxonomic group**	**Species name**	**Concentration (cell mL^{-1}, depth 0 m)**						
Diatom	*Bacteriastrum parallelum*	89	93	557	100	44	22	0
Diatom	Centric diatoms < 5 µm	22	232	93	55	0	44	0
Diatom	*Cerataulina pelagica*	22	0	0	0	44	210	11
Diatom	*Chaetoceros minimus*	0	0	0	0	44	22	0
Diatom	*Chaetoceros simplex*	421	604	1858	244	22	33	0
Diatom	*Chaetoceros* spp.	510	743	21928	6115	288	155	11
Diatom	*Chaetoceros tenuissimus*	1861	4832	24158	1163	22	89	66
Diatom	*Chaetoceros throndsenii*	1972	2973	6597	55	0	0	0
Diatom	*Cyclotella atomus* var. *gracilis*	155	418	279	0	0	0	0
Diatom	*Cyclotella* spp.	66	325	372	0	22	22	11
Diatom	*Cylindrotheca closterium*	709	1069	2973	521	465	277	155
Diatom	*Fragilariopsis pseudonana*	0	0	0	0	0	22	0
Diatom	*Leptocylindrus danicus*	66	46	0	0	66	122	222
Diatom	*Minutocellus polymorphus*	0	0	650	609	377	321	0
Diatom	Pennate diatoms < 10 µm	0	0	0	11	0	0	11
Diatom	Pennate diatoms > 10 µm	0	0	0	0	0	11	0
Diatom	*Proboscia alata*	0	0	0	11	0	0	0
Diatom	*Pseudo-nitzschia delicatissima*	0	0	0	0	44	0	11
Diatom	*Pseudo-nitzschia galaxiae*	332	929	186	233	44	277	22
Diatom	*Pseudo-nitzschia multistriata*	332	46	0	11	22	0	0
Diatom	*Skeletonema menzelii*	0	0	0	0	0	0	11
Diatom	*Skeletonema pseudocostatum*	798	11103	1580	122	0	11	0
Diatom	*Thalassiosira* spp.	44	46	186	499	177	188	11
Dinoflagellate	*Calciodinelloideae* n.d.	44	93	186	0	22	0	0
Dinoflagellate	*Lessardia elongata*	22	0	0	0	0	0	0
Dinoflagellate	Naked dinoflagellates < 15 µm	886	929	743	233	155	66	78
Dinoflagellate	Naked dinoflagellates > 15 µm	89	46	0	33	0	0	0
Dinoflagellate	*Oxytoxum variabile*	0	0	0	0	0	11	0
Dinoflagellate	*Prorocentrum donghaiense*	0	0	93	0	0	0	0
Dinoflagellate	*Protoperidinium diabolus*	22	0	0	0	0	0	0
Dinoflagellate	Thecate dinoflagellates < 15 µm	111	93	0	0	22	0	0
Dinoflagellate	Thecate dinoflagellates > 15 µm (heterotroph)	0	0	0	11	0	0	0
Coccolithophore	*Calciopappus caudatus*	0	0	0	0	22	0	0
Coccolithophore	*Emiliania huxleyi*	0	93	0	11	22	33	11
Coccolithophore	Undetermined coccolithophores	399	650	93	44	89	111	0
Other flagellate	*Chlamydomonas* spp.	0	0	0	11	0	0	0
Other flagellate	Coccoids	0	0	0	0	111	0	11
Other flagellate	*Dinobryon faculiferum*	44	0	0	78	22	22	0
Other flagellate	*Diplostauron* cf. *elegans*	0	93	279	0	0	0	0
Other flagellate	*Eutreptiella* spp.	0	139	0	0	0	11	0

Other flagellate	Heterotroph flagellates	22	0	93	100	111	210	33
Other flagellate	*Ollicola vangoorii*	133	139	557	122	44	33	0
Other flagellate	*Pachysphaera* spp.	0	0	0	0	0	0	11

Table S4 (cont'd)

Other flagellate	*Paulinella ovalis*	89	93	0	0	0	0	0
Other flagellate	*Pseudoscourfieldia marina*	465	976	186	89	89	78	11
Other flagellate	*Pyramimonas* spp.	111	0	0	66	0	0	0
Other flagellate	*Tetraselmis* spp.	44	0	0	0	22	0	0
Other flagellate	Undetermined cryptophyceans < 10 µm	1130	418	279	122	89	22	22
Other flagellate	Undetermined phytoflagellates < 10 µm	11743	26899	29547	5694	9683	3966	2980
Other flagellate	Undetermined prasinophyceae	22	0	0	0	0	0	0
	**Phytoplankton group**	**Biomass (µg C L^{-1}, depth 0 m)**						
	Diatoms	78.5	307.9	432.8	94.2	35.2	84.8	21.0
	Dinoflagellates	234.2	172.1	185.0	60.9	27.3	5.1	5.4
	Coccolithophores	13.0	23.3	3.0	1.7	3.6	4.3	0.2
	Other flagellates	85.1	166.7	185.6	35.7	52.5	24.4	15.8
	Total phytoplankton	410.7	670.0	806.4	192.5	118.7	118.6	42.4
		**Diversity - Fisher's alpha**						
	Total phytoplankton	3.8	3.0	2.2	3.4	3.6	3.8	2.8
		**Chlorophyll *a* (µg L^{-1}, 0-2 m, average value)**						
		5.64	5.69	13.51	3.40	1.08	0.85	0.49

Tables S5. Data of phytoplankton abundance, biomass, diversity plus chl *a* from station LTER-MC and used to produce elaborations in Fig. 8b.

		Date			
		9.12.09	16.12.09	21.12.09	29.12.09
Taxonomic group	Species name	Concentration (cell mL^{-1}, depth 0 m)			
Diatom	*Bacteriastrum furcatum*	0	0	0	5
Diatom	*Bacteriastrum parallelum*	0	0	0	1
Diatom	Centric diatoms < 5 µm	46	36	7	4
Diatom	*Cerataulina pelagica*	0	0	0	4
Diatom	*Chaetoceros contortus*	0	0	0	8
Diatom	*Chaetoceros curvi-curv*i	0	0	0	1
Diatom	*Chaetoceros curvisetus*	0	13	0	1
Diatom	*Chaetoceros lauderi*	3	0	0	0
Diatom	*Chaetoceros minimus*	20	26	0	0
Diatom	*Chaetoceros socialis*	705	537	427	16
Diatom	*Chaetoceros* spp.	0	43	7	0
Diatom	*Chaetoceros tenuissimus*	0	0	0	13
Diatom	*Cyclotella* spp.	3	0	0	0
Diatom	*Cylindrotheca closterium*	13	0	7	5
Diatom	*Guinardia striata*	3	0	3	0
Diatom	*Leptocylindrus danicus*	46	49	0	0
Diatom	*Leptocylindrus mediterraneus*	7	0	0	0
Diatom	*Leptocylindrus minimus*	7	0	0	0
Diatom	Pennate diatoms > 10 µm	3	0	0	5
Diatom	*Plagiotropis* cf. *lepidoptera*	10	0	0	0
Diatom	*Pleurosigma* spp.	0	0	0	0
Diatom	*Proboscia alata*	0	0	0	0
Diatom	*Pseudo-nitzschia delicatissima*	3	7	0	0
Diatom	*Pseudo-nitzschia fraudulenta/subfraudulenta*	13	0	0	0
Diatom	*Pseudo-nitzschia galaxiae*	7	3	3	3
Diatom	*Pseudo-nitzschia galaxiae* "small morphotype"	0	0	0	3
Diatom	*Pseudo-nitzschia multistriata*	7	0	0	0
Diatom	*Pseudo-nitzschia pseudodelicatissima*	26	3	13	0
Diatom	*Skeletonema menzelii*	79	43	7	0
Diatom	*Thalassionema bacillare/frauenfeldii*	0	0	0	5
Diatom	*Thalassionema nitzschioides*	13	0	0	0
Diatom	*Thalassiosira* spp.	63	23	3	0
Dinoflagellate	*Karenia* spp.	0	0	1	0
Dinoflagellate	Naked dinoflagellates < 15 µm	40	16	14	9
Dinoflagellate	Naked dinoflagellates < 15 µm (heterotroph)	0	0	0	0
Dinoflagellate	Naked dinoflagellates > 15 µm	0	3	4	1
Dinoflagellate	*Oxytoxum variabile*	0	0	1	1
Dinoflagellate	*Prorocentrum triestinum*	3	0	0	0
Dinoflagellate	Thecate dinoflagellates < 15 µm	7	0	1	1
Dinoflagellate	Thecate dinoflagellates > 15 µm	3	0	0	0
Coccolithophore	*Acanthoica quattrospina*	0	0	1	0
Coccolithophore	*Algirosphaera robusta*	0	0	0	1

Table S5 (cont'd)

Coccolithophore	*Calciopappus caudatus*	3	3	0	0
Coccolithophore	*Coronosphaera mediterranea*	0	0	1	0
Coccolithophore	*Emiliania huxleyi*	89	72	42	26
Coccolithophore	*Ophiaster* spp.	0	0	0	1
Coccolithophore	*Rhabdosphaera clavigera*	0	0	3	0
Coccolithophore	*Sphaerocalyptra quadridentata*	0	3	0	0
Coccolithophore	*Umbellosphaera* spp.	3	0	0	0
Coccolithophore	Undetermined coccolithophores	10	10	0	0
Other flagellate	*Dictyocha fibula*	0	0	1	0
Other flagellate	Heterotroph flagellates	7	10	3	0
Other flagellate	*Leucocryptos marina*	0	0	1	3
Other flagellate	*Meringosphaera mediterranea*	0	0	1	0
Other flagellate	*Ollicola vangoorii*	13	0	1	1
Other flagellate	*Paulinella ovalis*	0	7	8	12
Other flagellate	*Phaeocystis* spp.	3	3	0	0
Other flagellate	*Pyramimonas* spp.	10	0	1	0
Other flagellate	*Rhizomonas setigera*	33	0	0	0
Other flagellate	Undetermined cryptophyceans < 10 µm	49	30	5	33
Other flagellate	Undetermined phytoflagellates < 10 µm	774	636	427	286
Other flagellate	Undetermined phytoflagellates > 10 µm	0	0	1	0
	**Phytoplankton group**	**Biomass (µg C L^{-1}, depth 0 m)**			
	Diatoms	28.9	17.2	11.6	4.1
	Dinoflagellates	6.7	3.8	5.1	1.9
	Coccolithophores	2.5	2.2	1.3	0.7
	Other flagellates	5.5	3.8	2.8	2.1
	Total phytoplankton	43.6	27.0	20.9	8.8
		**Diversity - Fisher's alpha**			
	Total phytoplankton	6.0	3.6	5.4	6.0
		**Chlorophyll *a* (µg L^{-1}, 0-2 m, average value)**			
		3.09	2.31	1.24	0.86

Tables S6. Data of phytoplankton abundance, biomass, diversity plus chl *a* from station LTER-MC and used to produce elaborations in Fig. 8c.

		Date			
		**28.7.09**	**4.8.09**	**11.8.09**	**18.8.09**
**Taxonomic group**	**Species name**	**Concentration (cell mL^{-1}, depth 0 m)**			
Diatom	Bacteriastrum parallelum	836	1115	10	66
Diatom	Centric diatoms < 5 µm	93	0	3	177
Diatom	Cerataulina pelagica	0	139	82	44
Diatom	Chaetoceros contortus	0	0	26	0
Diatom	Chaetoceros curvi-curvi	0	0	0	22
Diatom	Chaetoceros simplex	1115	2880	63	399
Diatom	Chaetoceros spp.	2973	4739	82	5074
Diatom	Chaetoceros tenuissimus	3902	7062	10	1485
Diatom	Cyclotella atomus var. gracilis	186	0	0	0
Diatom	Cylindrotheca closterium	1672	743	26	1174
Diatom	Dactyliosolen blavyanus	0	0	0	22
Diatom	Dactyliosolen fragilissimus	0	0	13	0
Diatom	Leptocylindrus danicus	4739	5621	1090	4675
Diatom	Lioloma spp.	0	0	3	0
Diatom	Proboscia alata	0	93	7	0
Diatom	Pseudo-nitzschia galaxiae	10035	325	16	155
Diatom	Pseudo-nitzschia pseudodelicatissima	0	46	270	155
Diatom	Skeletonema menzelii	15889	10592	33	2437
Diatom	Skeletonema pseudocostatum	929	139	0	66
Diatom	Thalassiosira spp.	186	0	0	22
Dinoflagellate	Calciodinelloideae n.d.	0	0	3	44
Dinoflagellate	Lessardia elongata	0	0	3	0
Dinoflagellate	Naked dinoflagellates < 15 µm	0	46	30	133
Dinoflagellate	Naked dinoflagellates > 15 µm	93	0	7	22
Dinoflagellate	Protoperidinium spp.	93	0	0	0
Dinoflagellate	Thecate dinoflagellates < 15 µm	0	0	3	0
Coccolithophore	Calciopappus caudatus	0	46	3	0
Coccolithophore	Calciosolenia brasiliensis	0	0	0	22
Coccolithophore	Emiliania huxleyi	0	0	0	44
Coccolithophore	Undetermined coccolithophores	0	186	10	44
Other flagellate	Dinobryon faculiferum	93	93	3	22
Other flagellate	Diplostauron cf. elegans	93	0	0	0
Other flagellate	Heterotroph flagellates	0	0	23	111
Other flagellate	Leucocryptos marina	93	46	0	133
Other flagellate	Ollicola vangoorii	372	186	3	0
Other flagellate	Pachysphaera spp.	0	0	0	22
Other flagellate	Pseudoscourfieldia marina	186	139	20	22
Other flagellate	Pyramimonas spp.	0	46	0	0
Other flagellate	Tetraselmis spp.	0	0	3	0
Other flagellate	Undetermined cryptophyceans < 10 µm	1022	279	46	177
Other flagellate	Undetermined cryptophyceans > 10 µm	0	0	3	0
Other flagellate	Undetermined phytoflagellates < 10 µm	15145	8037	1166	9239

Table S6 (cont'd)

Phytoplankton group	Biomass (µg C L^{-1}, depth 0 m)			
Diatoms	637.6	672.4	109.8	439.7
Dinoflagellates	192.6	3.2	10.1	56.8
Coccolithophores	0.0	6.6	0.4	5.0
Other flagellates	103.3	47.1	7.3	53.3
Total phytoplankton	933.5	729.4	127.5	554.8
	**Diversity - Fisher's alpha**			
Total phytoplankton	2.0	2.3	4.6	3.1
	**Chlorophyll *a* (µg L^{-1}, 0-2 m, average value)**			
	4.44	5.31	0.78	3.01

Supplementary Note

VPPs zonal and meridional spreading

We computed the spatial variance of VPP origin zone at the end of backward trajectories (zonal and meridional spreading hereinafter) following Eq (3) reported in the Methods section. Figure S1 shows that both zonal and meridional spreading scored low values (< 150 m^2) for almost all coastal sectors, owing to the typical current speeds in the GoN (in the range of 10 - 25 cm s^{-1} all year round Figs. 2 in the main text). VPPs coming from the offshore origin zone present highest spreading values (in the range 100-250 m^2) in both directions compared to coastal origin ones. Only VPPs coming from the coastal sector 4 spread along the zonal direction up to 650 m^2 from the VPPs centre mass. This exception may be due to the frequent occurrence of a coastal cyclonic gyre (not shown) favouring particle dispersion from the coastline of sector 4 toward LTER-MC.

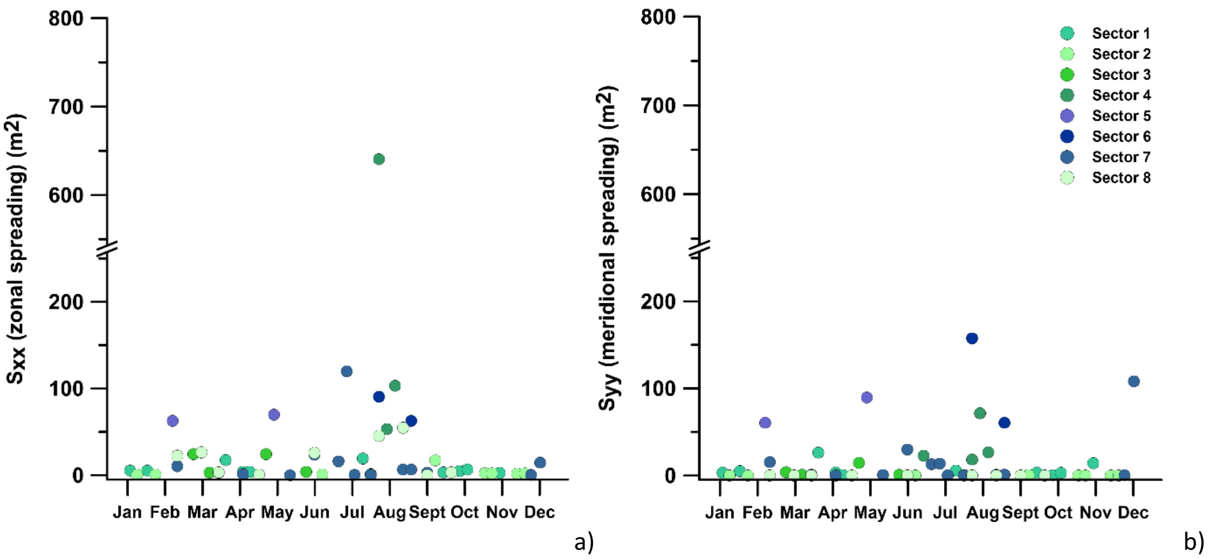

Supplementary Figure 1: Spatial variance of VPPs origin zone at the end of backward trajectories, grouped for the defined eight sectors. **a)** spreading along the zonal direction (x direction); **b)** spreading along meridional direction (y direction). Different colour indicate the origin sector of the VPPs (see also Fig. 3a in the main text).
